# Encore: Behavioural animal models of stress, depression and mood disorders

**DOI:** 10.3389/fnbeh.2022.931964

**Published:** 2022-08-08

**Authors:** Aleksa Petković, Dipesh Chaudhury

**Affiliations:** Laboratory of Neural Systems and Behaviour, Department of Biology, New York University Abu Dhabi, Abu Dhabi, United Arab Emirates

**Keywords:** animal model, behaviour, depression, mood, stress, rodent, disorder, symptom

## Abstract

Animal studies over the past two decades have led to extensive advances in our understanding of pathogenesis of depressive and mood disorders. Among these, rodent behavioural models proved to be of highest informative value. Here, we present a comprehensive overview of the most popular behavioural models with respect to physiological, circuit, and molecular biological correlates. Behavioural stress paradigms and behavioural tests are assessed in terms of outcomes, strengths, weaknesses, and translational value, especially in the domain of pharmacological studies.

## Major depressive disorder

Neuropsychiatric disorders affect both cognition and behaviour. Their primary causes are maladaptive responses of the central nervous system. In 2017, an estimated 971 million individuals worldwide suffered from mental disorders with depressive disorders accounting for 27% and anxiety disorders accounting for 29% of the estimated prevalence (James et al., 2018). These disorders are accompanied by alterations in individuals’ perception of social reality, adaptive ability, and executive functioning (Ferreri et al., 2011; Millan et al., 2012; Weightman et al., 2014; Solé et al., 2017; Italia et al., 2020). Currently, there are no obvious traceable structural and molecular correlates that uniquely map to specific disorders (Nestler and Hyman, 2010). A unique issue related to the treatment of neuropsychiatric disorders is frequent symptom relapse following drug withdrawal (Hyman, 2014).

Major depressive disorder (MDD) belongs to a category of heterogeneous disorders converging on symptomatology, but diverging in causes (Wallace et al., 2020). Longitudinal studies suggest that current prevalence rates could be 50% higher than officially reported (Toseeb et al., 2014). In the United States, this would amount to a projected lifetime incidence of 20–25%, with the risk of MDD increasing with decreasing age, indicating the significance of the generational factor (Ferrari et al., 2013). A recent meta-analysis revealed a 27.6% increase in worldwide cases in 2020 due to COVID-19 alone (Santomauro et al., 2021). Two-thirds of those that commit suicide suffered from MDD (Van Dam et al., 2017). The heritability of depressive disorders is high at 37% implying underlying genetic differences that drive stress vulnerability (Whiteford et al., 2013). However, the MDD onset requires a trigger from environmental factors. Even though a significant portion of the population is treatment-resistant, no major advances have been achieved concerning the prevention and discovery of more universally applicable treatments (Czéh et al., 2016). Around 50% of patients are resistant to treatment with classical monoamine-increasing antidepressants (Berton et al., 2006). Yet, most MDD treatments still aim to increase monoamine abundance, even though the monoamine theory of depression has been abandoned over two decades ago (Chaudhury et al., 2015; Czéh et al., 2016).

Diagnosis of MDD, according to the DSM-5 guidelines, requires the presence of five or more symptoms for 2 weeks, with at least one of the symptoms being either depressed mood or loss of pleasure and interest (American Psychiatric Association [APA], 2013). Other related symptoms include the following: significant appetite disturbance and weight change, insomnia or hypersomnia, psychomotor agitation or retardation, fatigue and loss of energy, weakened attention and cognitive abilities, indecisiveness, feelings of worthlessness or excessive guilt, and recurrent intrusive thoughts of death and suicide (American Psychiatric Association [APA], 2013). Thus, MDD is often accompanied by bidirectional disruptions (Alcantara et al., 2017). Besides showcasing heterogeneity of the disorder, this observation further aggravates model establishment.

Comorbidity of MDD with other disorders is high. Based on NCS-R estimations, year-long and lifetime comorbidity rates between depressive disorders and anxiety disorders are 67.8 and 59.2%, respectively (Van Dam et al., 2017). The lifetime comorbidity rate between depressive disorders and other neuropsychiatric disorders is 72.1%, which is significant given that comorbidity with other disorders predicts attenuated antidepressant treatment response (Van Dam et al., 2017). Patients with MDD are expected to live 5–10 years less, mostly due to the development of serious somatic conditions (Van Dam et al., 2017). Other risk factors include sex where women have a two-fold higher risk of MDD (Kessler, 1993; Soldin and Mattison, 2009) and lower socioeconomic status which predicts disproportionally higher incidence, prevalence, and persistence of the disorder (Lorant, 2003).

## Approaches and challenges in animal modelling

Modelling of human disease is in itself challenging. However, the additional difficulty in modelling psychiatric disorders lies in the subjective nature of many neuropsychiatric symptoms and the lack of clearly defined and sensitive diagnostic criteria (Nestler and Hyman, 2010; Czéh et al., 2016). As cognitive and emotional symptoms cannot be definitively established in animals, bluntly asserting particular symptomatology to animals represents an unjustified intellectual leap (Geyer and Markou, 1995; Nestler and Hyman, 2010). This is the reason behind the recent subtle change in the field’s terminology, with a shift toward the use of more hypothetical language when describing an animal model (Nestler and Hyman, 2010; Italia et al., 2020). Neuropsychiatric models can be developed through (1) a combination of plausible risk factors or (2) the establishment of a reasonable degree of neural and behavioural pathologies that correspond to the human disease. The intended scope of the model ought to dictate the set of criteria that the model needs to fulfil for it to be considered a valid model of human disease. Thus, it is beneficial to first assess the spectrum of purposes for which animal models are used. Historically, there have been four general approaches to animal modelling: early modelling, pharmacological, symptoms-oriented, and construct modelling (Geyer and Markou, 1995).

Early modelling approaches attempted to fully reproduce human disorders, despite the subjective nature of the symptoms (Crawley, 2000). Apparent similarities are still often utilized to justify the use of some common animal models. Continuous revision of diagnostic labels, alongside recently emerging understanding of disease heterogeneity, rendered such approaches obsolete. Consequentially, pharmacological studies suffered from the use of models with low construct and predictive validity (Markou et al., 2009; Nestler and Hyman, 2010). The pharmacological approach focuses on novel treatment discovery by investigating compounds that are structurally and mechanistically similar to currently used drugs (Hyman, 2014; Czéh et al., 2016). This approach has not led to the discovery of truly novel classes of drugs for current treatment-resistant endophenotypes (Matthysse, 1986; Hyman, 2014; Czéh et al., 2016). One explanation is likely that most behavioural screens assess drug efficacy shortly after acute administration, whereas most current antidepressants achieve relevant clinical effects after weeks of therapy (Czéh et al., 2016). The efficacy of many antidepressants has been brought into question, even though they passed the initial screening in animals and were reported to have substantial benefits (Ioannidis, 2008).

Currently, the most widely utilised approach is to recapitulate particular traits and symptoms that comprise an endophenotype rather than attempt to model the whole disorder (Cryan and Slattery, 2007; Czéh et al., 2016; Anderzhanova et al., 2017). This “symptoms-oriented” approach developed with a growing drift in the early 2000s away from attempting to treat the whole disorder toward treating individual symptoms. In neuropsychiatric research, endophenotypes represent specific behavioural, anatomical, physiological, molecular, or biochemical clusters of symptoms (Gottesman and Gould, 2003; Hasler et al., 2006). Modern symptoms-oriented and construct approaches are based on functional similarity that takes into into account the evolutionary divergence of adaptive behaviours (Cenci et al., 2002). However, distinct disorders can have similar primary symptoms, which makes experimental conclusions in the animal context uncertain at best, even in the case of translatable core symptoms (Matthews et al., 2005; Czéh et al., 2016; Anderzhanova et al., 2017). Future research on the high comorbidity rates between different disorders will further reveal diverging causative processes behind apparently similar symptoms (Van Dam et al., 2017). Limiting the scope of symptomatology being captured by the model increases the confidence in its *trans*-species validity which is main advantage of the symptoms-oriented approach (Geyer and Markou, 1995). However, this approach does not allow for the study of common causes and interactions between symptoms.

Hence, capturing the full complexity of a disorder requires theorising diagnostic constructs hypothesised to be affected in animals. Such construct-oriented approach necessitates the equivalence of the causes of particular symptoms in animals and humans. Thus, this approach moved away from modelling symptoms toward modelling causes. The bidirectional communication between basic and clinical research on underlying constructs and criteria for the model establishment, which is currently limited as evident through arbitrary and sometimes non-empirical changes in disorder labels and diagnostic guidelines (American Psychiatric Association [APA], 2013; Murphy and Hallahan, 2016). The continuous revision of diagnostic criteria is due to the lack of understanding that basic research should provide. Consequentially, there is considerable difficulty in translating the basic research findings into tangible clinical treatments. Various neuropsychiatric symptoms can be modelled in animals, but it is uncertain how these add up to a particular human disorder. This issue is also prevalent in diagnosing neuropsychiatric disorders in humans where an arbitrary number of symptoms needs to be displayed for the disorder to be diagnosed and where some symptoms are given precedence over others (American Psychiatric Association [APA], 2013). Since neuropsychiatric disorders can be highly heterogeneous in their causes and symptoms, neuropsychiatry requires basic research to provide greater insight into the formation of the disease in order to develop more objective diagnostic procedures in humans (López-León et al., 2008; Bosker et al., 2011; Cross-Disorder Group of the Psychiatric Genomics Consortium, 2013; Wray et al., 2018). Animal models are unlikely to ever mirror the full extent of human neuropsychiatric disorders, and even those symptoms that can be ascertained with some confidence might not have a direct equivalence with human symptoms (Nestler and Hyman, 2010; Italia et al., 2020). Instead of serving as defining features of the disease, symptoms can better serve as an identifying principle for genomic and molecular studies (Manchia et al., 2013; Gade et al., 2015; Ohi et al., 2015; Papassotiropoulos and de Quervain, 2015). In that sense, construct modelling has been most fruitful as it is based on an informational feedback loop between basic research and clinical applications. The broad consensus is that the study of complex and heterogeneous psychiatric disorders requires prior study of normal social, reward, mood, circadian, and sleep physiology (Wirz-Justice, 2006; Proulx et al., 2014; Golden et al., 2019; Modi and Sahin, 2019; Radwan et al., 2019, 2021).

### Choosing the animal model

#### Species

Initially, rats were preferred over other rodents due to their stronger performance on conditioning and cognitive tasks, and their robustness and size which allowed easier invasive experimental manipulation (Cryan and Holmes, 2005). Powerful and diverse genetic manipulations that enabled an easier study of mammalian gene function shifted the bias toward mice (Rossant and McKerlie, 2001; Cryan and Holmes, 2005; Wahlsten, 2011). Though much less sensitive to environmental effects than rats, mice exhibit less uniform responses, necessitating bigger sample sizes (Castagné et al., 2010). However, the ease with which mice are bred and maintained allows for increased sample sizes and greater statistical power. Their 2-year lifespan allows for long-term studies of chronic conditions. Importantly, the neurological complexity of mice is similar enough to humans to potentially mirror human endophenotypes. Mice models share many biochemical and physiological pathways with humans (Rossant and McKerlie, 2001). Mice and humans share evolutionary conserved stress-coping mechanisms, and they express genes with similar functions (Mouse Genome Sequencing Consortium, 2002; Italia et al., 2020). Important traits such as disease susceptibility are inherited in a similar manner (Rossant and McKerlie, 2001). Currently, thousands of strains and transgenic mouse lines are available. Initially, genetic variation between different strains prompted investigations into genetic and molecular vulnerability factors. This remains one of the focal points of neuropsychiatric research (Pothion et al., 2004; Einat, 2007; Zimmermann et al., 2016). Strain investigations have recently been more focussed toward behavioural and neurophysiological stress responses.

Despite stark similarities between rodent and primate physiology, some researchers argue that non-human primate models should exclusively guide neuropsychiatric and pharmacological research, whereas the rodent models remain suitable for genetic and molecular investigations (Cenci et al., 2002). Although cerebrocortical differences between rodents and humans limit the modelling of higher cognition, subcortical structures, which serve as the primary origin of maladaptive stress-related responses, are almost identical (de Kloet et al., 2005; Joëls and Baram, 2009). These midbrain centres regulate several autonomic functions, such as stress and reward responses, which are disrupted in neuropsychiatric disorders (Joëls and Baram, 2009; Cho et al., 2021; Metzger et al., 2021). Although abstract thought patterns and complex emotions remain elusive in rodents, the activity of subcortical circuitry and its effect on behaviour can reveal much about shared underlying causative processes. Low maintenance of rodents and rising ethical issues sidelined primate models (Italia et al., 2020). In 2015, 32% of neuroscience studies were conducted on rodents, with only 1% of research involving other species (Ambigapathy et al., 2015; Geva-Sagiv et al., 2015; Mello and Clayton, 2015; Keifer and Summers, 2016). Many earlier advances have been achieved using invertebrate and ‘exotic’ vertebrate models, despite their neglect in the last two decades (Manger, 2008). Furthermore, many evolutionarily more distant species display highly homologous functions which are better or as good proxy for human functioning compared to those found in rodents, such as is the case with spatial cognition in bats or study of vocal learning and social behaviour in songbirds (Ambigapathy et al., 2015; Geva-Sagiv et al., 2015; Mello and Clayton, 2015). Understanding maladaptive behaviours requires an understanding of evolutionary mechanisms and causes behind the altered adaptive behaviours.

#### Strain

Large behavioural and physiological differences exist between rodent strains. Significant differences have been observed in motor skills (Brooks et al., 2004), cognitive and learning abilities (Baron and Meltzer, 2001; Brooks et al., 2005; Codita et al., 2012), and baseline anxiety-like and depressive-like behaviours (Pothion et al., 2004; Bothe et al., 2005; Zimmermann et al., 2016). Some strains differ in sensory processing which directs subsequent behaviour (Bailey et al., 2006). Readily available inbred strains display minimal variability, whereas the ease of genetic manipulation allows for the research of specific allelic variants which are thought to contribute to a particular disorder (Italia et al., 2020). Inbred strains have been outside their natural environment for several generations and consequentially do not exhibit natural genetic and behavioural diversities, providing more uniform responses (Keifer and Summers, 2016).

#### Sex

Although there are significant differences in drug metabolism and neurophysiological functioning between sexes, such as drug clearance rate and hypothalamic–pituitary–adrenal axis (HPA) responsiveness, most neuropsychiatric research was almost exclusively performed on male rodents (Goel and Bale, 2009; Soldin and Mattison, 2009; Czéh et al., 2016; Heck and Handa, 2019). Women are two times as likely to develop stress-related pathologies, such as MDD and anxiety—outcomes, which may be attributed to sex steroids and sex-specific development of the brain (Naninck et al., 2011; Czéh et al., 2016). Only recently has this disparity in research been recognised (Dalla et al., 2010; Kokras and Dalla, 2014; Walker et al., 2022). Recent rodent studies confirm earlier clinical data that males and females exhibit different vulnerabilities and responses to stress and treatment (Soldin and Mattison, 2009; Dalla et al., 2010; Balhara et al., 2012; Kokras and Dalla, 2014; Bangasser and Wicks, 2017).

### Validity of animal models

All animal models are based on the assumption that there is an evolutionary homology between the behavioural, physiological, and molecular traits of human disorders and the animal model (Geyer and Markou, 1995; Cenci et al., 2002). Models are assessed in terms of reliability, consistency, and validity (Chadman et al., 2009; Nestler and Hyman, 2010). Reliability and consistency of tested behaviour are defined, respectively, as a positive correlation between multiple and within one measurement.

Construct validity refers to the methods used to establish the model. Ideally, the pathophysiological processes that led to disease onset in humans would be replicated on the neural and behavioural levels in the model (Chadman et al., 2009; Nestler and Hyman, 2010; Belzung and Lemoine, 2011). It is important to note that similar aetiology does not imply similar symptomatology and *vice versa* (Czéh et al., 2016). Almost all current models are based on causative processes, although a linear relationship between cause and the outcome is uncommon in psychiatry (Czéh et al., 2016; Anderzhanova et al., 2017). Most current models only utilise environmental factors, such as stress exposure, to induce disorder-like behaviour in rodents (Nestler and Hyman, 2010; Italia et al., 2020). First, it is unclear what constitutes a valid phenotype, mostly due to variations in definitions, stress type, and intensity. Second, identical environmental factors can contribute to the development of multiple disorder-like phenotypes. It is unknown to which degree these highly heterogeneous disorders have a common neurological basis in humans, which further limits the construct validity of current stress-based models (Davidson et al., 2001; Chadman et al., 2009; Italia et al., 2020).

Face validity is the measure of similarity between anatomical, biochemical, neuropathological, and behavioural markers in human patients and the animal model (van der Staay et al., 2009). There are very few, if any, reliable biomarkers of common neuropsychiatric disorders in humans (Anderzhanova et al., 2017). Thus, human-like behaviours in animals are still lacking definitive face validity. Due to species differences, no model is expected to capture all the features of human disease or even to fully reproduce single symptoms (Czéh et al., 2016). Most models display only a subset of symptoms and are void of typical comorbidities observable in humans (Anderzhanova et al., 2017).

The predictive validity corresponds to the equivalence of the treatment outcomes in human patients and the animal model (Nestler and Hyman, 2010; Wahlsten, 2011; Gururajan et al., 2019). Mechanisms of action of major classes of drugs are commonly investigated only after they are found to be antidepressant in humans (Nestler and Hyman, 2010). Paradoxically, rescuing animals through the action of these commonly used drugs, mostly monoaminergic antidepressants and anxiolytics, is commonly used to validate models which are predominantly supposed to guide research on treatment-resistant patients (Cryan and Slattery, 2007; Italia et al., 2020). Furthermore, treatment timecourse is rarely taken as a controlled variable in animal research, which lowers actual predictive validity (Czéh et al., 2016). Currently, no single model has optimal strength in all three validity types (Belzung and Lemoine, 2011).

## Modelling symptoms

Behavioural tests are one way to probe the rodent phenotype and are thought to model MDD symptoms (Anderzhanova et al., 2017; Planchez et al., 2019). Most behavioural tests are being standardised because environmental parameters within different laboratories can interact with genotype in unpredictable and significant ways (Tucci et al., 2006). For example, strain performance in different behavioural assays can strongly depend on the particular apparatus configuration and testing timing (Wahlsten et al., 2003). Standardisation filters out deleterious practices which do not converge across the field (Wahlsten, 2011). The persisting outcome discrepancies can then be further investigated as biologically meaningful (Würbel, 2000, 2002). The potential benefits of standardising all testing parameters are important to consider since seemingly unimportant factors can exert a major influence on behaviour (Cryan and Slattery, 2007; Wahlsten, 2011). Finally, the most commonly used behavioural tests in mice were originally designed for use in rats which is likely one of the factors influencing low study reproducibility, alongside variations in the testing protocols (Czéh et al., 2016). Table 1 gives an overview of the main behavioural tests highlighting their strengths, weaknesses, and validity.

**TABLE 1:** Overview of main behavioural tests with respect to their strengths, weaknesses, and validity.

Behavioural test	Symptom	Strengths	Weaknesses	Validity		
				Construct	Face	Predictive
Sucrose preference test	Anhedonia	• Well established and validated • Small time investment • Minimal stress	• High variability in protocols • Very sensitive to environmental conditions • Low reproducibility	Strong	Very strong	Strong
Intracranial self-stimulation	Anhedonia	• Stable over time • Reliable	• Highly invasive • High time investment • Confounded by learning and memory deficits	Strong	Strong	Strong
Forced swim test	Behavioural despair	• Very sensitive to different classes of classic antidepressants • High reliability and reproducibility • Small time investment	• Rarely detects novel antidepressants • Extremely sensitive to environmental conditions • Highly strain-dependent • Test-retest effects • Confounded; Alternative explanations exist • Stressful • Hypothermia	Very weak	Weak	Strong for classic antidepressants Weak for novel antidepressants
Tail suspension test	Behavioural despair	• Very sensitive to different classes of classic antidepressants • High reliability and reproducibility • Small time investment • No test-retest effect • Minimal stress	• Rarely detects novel antidepressants • Sensitive to environmental conditions • Highly strain-dependent	Further research required	Moderate	Strong for classic antidepressants Weak for novel antidepressants
Splash test	Apathy	• Small time investment • Minimal stress	• Sensitive to environmental conditions • Diverging results from across the paradigms • Not applicable in CSDS due to coat deterioration	Moderate	Moderate	Weak
Social interaction test	Sociability	• Well-established	• Possibly confounded in CSDS • Highly strain-dependent • Anxiogenic • Test-retest effects	Very strong	Moderate	Strong
Crowley’s sociability test	Sociability	• Well-established • Asses social memory and social affiliation	• Highly strain-dependent • Anxiogenic	Very strong	Strong	Strong

### Anhedonia

One of the two core symptoms of MDD is anhedonia—loss of interest in initiating and participating in activities that were previously enjoyable, or a deficit in the capacity to feel pleasure (American Psychiatric Association [APA], 2013; Alcantara et al., 2017). In rodents, anhedonia is modelled as a decreased drive for food, sex, and pleasure in general (Alcantara et al., 2017). Doubts have arisen about the usefulness of modelling anhedonia as it is not specific to MDD and does not necessarily accompany the disorder, as exemplified by its onset due to stimulant withdrawal (Matthews et al., 2005; Nestler and Hyman, 2010). Sucrose reinforcers under a progressive schedule can be used to assess the withdrawal-induced reward deficits (Cryan et al., 2002). However, because anhedonia is deemed as one of two core symptoms of MDD, the bulk of research is still directed at further investigating its causes for the purposes of modelling MDD (Nestler and Hyman, 2010; Planchez et al., 2019).

The sucrose preference test (SPT) is the most widely used behavioural assay for establishing anhedonia in rodents (Liu et al., 2018). It was initially used to investigate the gustatory system and motivated behaviours in stress-naive mice (Hasegawa and Tomita, 1986). The test consists of offering the rodent a choice of two bottles: one with water and the other with sucrose solution (Eagle et al., 2016; Liu et al., 2018). The solution concentration varies between 0.25 and 2% (Eagle et al., 2016; Liu et al., 2018). Commonly, a ratio of the consumed volume of sucrose solution and the total volume of liquid consumed is used as a relative measure of animal’s preference. Reporting the ratio accounts for the baseline differences in liquid consumption and provides a more stable readout than reporting absolute volumes (Liu et al., 2018). Rodents naturally have a strong tendency to consume sweet food and prefer them over other dietary sources. Different criteria for defining anhedonic behaviour are used, but a common arbitrary criterion is a decrease in sucrose preference to below 60% (Strekalova and Steinbusch, 2010). SPT exhibits very strong face validity as it reproduces one of the two core symptoms of MDD (Nestler and Hyman, 2010; Pollak et al., 2010; American Psychiatric Association [APA], 2013). Anhedonic response in SPT is the most commonly reported effect of chronic stress (Willner et al., 1992; Willner, 1997; Pothion et al., 2004; Krishnan et al., 2007). Any effect of chronic stress on SPT subsides significantly only after 3 weeks, implying stability and longevity of the anhedonic phenotype (Yan et al., 2010). Selective serotonin reuptake inhibitor (SSRI) citalopram, paroxetine, and fluoxetine prevent and reverse anhedonia under conditions of chronic stress (Strekalova et al., 2006; Casarotto and Andreatini, 2007; Liu et al., 2015; Alcantara et al., 2017). Similarly, anhedonia can be rescued by prolonged administration of tricyclic antidepressant (TCA) desipramine (Willner et al., 1987). Despite its clear predictive value, SPT is rarely used as an antidepressant screen.

There is high variability in the SPT protocols which led to low reproducibility across studies, especially in mice (Strekalova and Steinbusch, 2010; Liu et al., 2018). Significant challenges have been posed in the past by differential strain response, differences in handling, and environmental inconsistencies that produce unreliable behavioural output (Tordoff and Bachmanov, 2002; Pothion et al., 2004; Strekalova et al., 2011; Eagle et al., 2016). The composition of other dietary sources can strongly influence the preference magnitude and the development of sucrose consumption and palatability in adult rodents (Bertino and Wehmer, 1981; Tordoff and Rabusa, 1998). Mice are often neophobic to sucrose solution which can be solved by pretest exposure to sucrose (Strekalova and Steinbusch, 2010). However, prior exposure causes a ceiling effect in the preference, which can be prevented by gradually decreasing sucrose concentration (Strekalova et al., 2006; Strekalova and Steinbusch, 2010). Overall, SPT scores are not very robust as they depend on test duration, sucrose saturation, and relative time of sucrose solution introduction (Tordoff and Bachmanov, 2002; Strekalova and Steinbusch, 2010). It was found thatat least partly pre-existing differences, and not effects of stress itself, determine the onset of anhedonia in SPT which lowers contrust validity of the test (Strekalova et al., 2004; McArthur and Borsini, 2006). This might be useful as one important approach to studying stress-induced pathologies is understanding how stress interacts with pre-existing vulnerability. This remains an important and largely uninvestigated avenue. One attempt at minimising the methodological variability and logistical constraints includes a recent automatisation of the SPT protocol which provided robust results across depression induction models (Yin et al., 2021). Despite all the limitations, SPT remains the single best measure of anhedonia (Castagné et al., 2009).

Intracranial self-stimulation (ICSS) in rodents involves the same brain regions that are implicated in human depression, namely, the hippocampus and amygdala, which adds to its construct validity (Slattery et al., 2007). The electrodes are directly implanted into hedonic centres of the brain— commonly the medial forebrain bundle that contains dopaminergic projections from the ventral tegmental area (VTA) (Chaudhury et al., 2013; Alcantara et al., 2017; Planchez et al., 2019). The rodent learns that pressing a lever will cause pleasurable sensations due to the electric stimulation of dopamine release. Increments in the amount of current needed to invoke an operant response are reflective of the changes in reward threshold which is elevated in stress disorders (Alcantara et al., 2017). Some researchers consider it as a measure of hedonic response (Matthews et al., 2005; Alcantara et al., 2017) and others also a measure of motivation (Scheggi et al., 2018). A particular advantage of ICSS is that no stimulation tolerance forms and that the response remains stable over time, allowing for extended periods of continuous and accurate detection of the emergence of anhedonia and its remission under treatment (Markou and Koob, 1992; Kornetsky, 2004; Scheggi et al., 2018). Additionally, the response is independent of sensory input, satiation, and stress-induced anxiety, which increases reliability (Wise, 2005; Scheggi et al., 2018). The direct relationship between self-stimulation and anhedonia guarantees good face and construct validity (Markou and Koob, 1992; Pollak et al., 2010). The method has been validated in a range of chronic stress paradigms (Casarotto and Andreatini, 2007; Wiborg, 2013; Der-Avakian et al., 2014; Donahue et al., 2014). However, ICSS is a highly invasive technique as it requires implantation of stimulation electrodes, rendering it impractical for research with a large number of animals and stress models with relatively high mortality, such as chronic social defeat stress (CSDS). Furthermore, impairments of learning and memory strongly confound ICSS relative to other measures.

Additional measures of anhedonia in rodents include sexual behaviours (Alcantara et al., 2017). Sexual behaviours in male rodents are attenuated as a consequence of chronic stress (Willner, 2005). Many antidepressants further decrease sexual performance which corresponds to the experiences of some human patients undergoing chronic antidepressant treatment (Werneke et al., 2006). The use of sexual drive as a proxy for anhedonia is not widespread, predominantly because it depends on the proception of female rodents at the time of testing. To circumvent the issue of proception, females undergo surgical ablation of the ovaries and hormonal treatment as a part of the standard preparation method (Planchez et al., 2019).

Anhedonic behaviour has been chiefly associated with changes in the VTA and nucleus accumbens (NAc). For example, anhedonic response in SPT has been associated with maladaptations in the dopaminergic VTA-NAc projection (Eagle et al., 2016). Specifically, anhedonia is caused by local increase in cAMP response element-binding protein (CREB) in the NAc (Barrot et al., 2002). Physiologically, experience of reward attenuates with decreased NAc neuronal activity and dopamine release (Gold, 2015). Consistently, anhedonia is negatively correlated with the volume and responses in NAc of human patients (Pizzagalli et al., 2009). Natural rewards, including food preference, sexual behaviour, and self-stimulation, are also regulated by the ΔFosB levels in the NAc (Wallace et al., 2008). Additional regions associated with anhedonia are the medial prefrontal cortex (mPFC) and dorsolateral prefrontal cortex (Palazidou, 2012).

Overall, the most robust anhedonic responses are produced by chronic unpredictable mild stress (CUMS) (Schweizer et al., 2009; Wiborg, 2013). Previously, it was shown that amphetamine withdrawal can also induce an anhedonic response in ICSS (Cryan et al., 2003). This withdrawal effect is absent in the progressive-ratio sucrose-reward task (Russig et al., 2003). One possible reason is that, while ICSS assesses hedonic response, the progressive-ratio sucrose-reward task reflects motivation. Generally, it has been found that there is a dissociation between wanting (motivation to acquire the reward) and liking (hedonic response to the reward) (Barr and Phillips, 1998). It might well be the case that these have separate neural correlates. Even though the amphetamine-withdrawal was proposed as a possible model for studying comorbidity between depression and substance abuse, only a few studies followed up (Haj-Mirzaian, 2018). Anhedonia is a complex phenomenon where disruption can occur on the level of hedonic response itself, reward expectation and motivation to obtain the reward, and development and execution of a strategy to obtain the reward. Using different tests, such as SPT and ICSS, one goal of future studies should be to investigate whether different stress models lead to impairment at different or the same levels of hedonic response.

### Behavioural despair, fatigue, and helplessness

Other common symptoms of MDD are behavioural despair and helplessness. Behavioural despair in animals is often associated with two tests commonly used in depression research: the forced swim test (FST) and tail suspension test (TST) (Nestler et al., 2002; Can et al., 2011b,^2012^; Slattery and Cryan, 2012).

Forced swim test was developed to model rodent behaviour reminiscent of despair and predict the effects of novel antidepressants (Porsolt et al., 1977). Antidepressant screening remains its main purpose today (Slattery and Cryan, 2012). The standard testing procedure in rats consists of multiple drug administrations between two testing sessions, whereas mice were found to demonstrate a sufficient and stable level of immobility after single drug administration and FST exposure (Castagné et al., 2010). The test was developed based on the observations that rodents forced to swim without the possibility of escape assume complete immobility after the initial burst of struggling (Porsolt et al., 1977). In this state, the rodent only performs movements that help to maintain its head above the water (Biselli et al., 2021). Porsolt described this behaviour as a “state of despair” which is brought about by the animal’s “realisation” that escape is impossible (Porsolt et al., 1977).

Slight modifications from original Porsolt’s test made FST sensitive to SSRIs and allowed for more sophisticated differentiation of the various behavioural responses (Lucki, 1997; Cryan et al., 2002). Some antidepressants have specific effects on swimming, climbing, and sexually dimorphic head-shaking (Kelliher et al., 2003; Kokras et al., 2017; Biselli et al., 2021). The modified FST was shown to be sensitive to different classes of antidepressants (Cryan et al., 2002; Pollak et al., 2010). For example, SSRI, TCA, and catecholaminergic antidepressants decrease total immobility duration (Cryan et al., 2002). However, SSRI antidepressants increase swimming frequency and overall time spent swimming, whereas TCA and catecholaminergic drugs increase climbing frequency and duration (Lucki, 1997; Cryan and Lucki, 2000; Belovicova et al., 2017). In contrast to rats, mice only exhibit immobility (Can et al., 2012). Besides total immobility duration, another parameter measured in both rats and mice is immobility latency which is often taken as a critical feature in distinguishing between the antidepressant and stimulatory classes of drugs (Castagné et al., 2009). FST often requires additional behavioural confirmation due to many possible confounds, the most common being general disruption of locomotor abilities (Pollak et al., 2010; Can et al., 2011b). The major advantage of using FST is its low cost and time investment (Cryan et al., 2002; Yan et al., 2010). However, the test is extremely sensitive to a range of environmental factors (Petit-Demouliere et al., 2005; Bogdanova et al., 2013; Zhou, 2016).

The TST is similar in nature and effects to FST (Steru et al., 1985). The mouse is suspended by its tail and will initially try to escape the stressful situation. After some time, it becomes immobile. The main parameters measured are immobility time and latency to immobility. As in the FST, longer immobility periods are taken as symptoms of a depressive-like state which can be attenuated with antidepressant administration (Thierry et al., 1986; Ripoll et al., 2003; Teegarden, 2012). Particular types of movements, such as swinging and curling, can reliably be recorded using more sophisticated ethological software (Can et al., 2012). For example, noradrenaline reuptake inhibitors and SSRIs have been reported to selectively increase swinging, whereas opioid compounds selectively increased curling (Berrocoso et al., 2013). This indicates that swinging and curling might be selectively tied to monoamine and opioid transmission, respectively. One of the advantages of TST is that it can be repeated several times without the confounding test–retest effects and is more easily implemented than FST while remaining sensitive to all of the major classes of antidepressants (Cryan and Mombereau, 2004; Pollak et al., 2010). Contrary to FST, mice exposed to TST immediately resume normal levels of locomotor activity. Furthermore, no post-experimental procedure is required, such as warming up the animal (Castagné et al., 2010). Another major advantage is that TST avoids the risk of hypothermia and allows for the assessment of the strength and energy of the animal’s movements (Ripoll et al., 2003; Castagné et al., 2010; Belovicova et al., 2017). FST-induced hypothermia in mice is severe as it has been reported that after 6 min in 23°C warm water, the mouse’s body temperature drops by 6.5°C (Porsolt et al., 1977; Thierry et al., 1986). Hypothermia can be a confounding factor in antidepressant research due to its effects on drug metabolism (Thierry et al., 1986). Furthermore, the distressed behaviour that is evident during and after FST, but absent from TST, can affect subsequent tests, which is why FST is usually performed at the end of the behavioural test batteries. A much smaller dose of antidepressant is required for a significant reduction in immobility time in TST as compared to FST (Porsolt et al., 1977; Steru et al., 1985; Thierry et al., 1986). It is important to note that the pattern of drug response, and sensitivity differ between FST and TST over the same dose range (Yan et al., 2010).

The FST is commonly used in rats due to their swimming proficiency, whereas TST is more appropriate for in mice because of their smaller weight. It is expected that a rat will remain immobile for about two-thirds of a 5-min session in baseline FST, whereas the average immobility for mice is around 2 min in both FST and TST (Castagné et al., 2010). In FST, outbred mouse strains are more sensitive to antidepressants, whereas inbred strains display significantly smaller intragroup variability (Petit-Demouliere et al., 2005). Contrastingly, different inbred mouse strains display widely varying responses to different classes of antidepressants in TST (Bai et al., 2001; Ripoll et al., 2003; Crowley et al., 2005; Can et al., 2011a). For example, outbred NIH-Swiss strain and inbred C57BL/6JRj strains showed decreased immobility following administration of TCAs, SSRIs, and dopamine reuptake inhibitors, whereas outbred NMRI and inbred DBA/2 strains were selectively sensitive to SSRIs (Ripoll et al., 2003). Similar differences in inbred strains are commonly observed in FST (Jacobson and Cryan, 2007; Funayama et al., 2022). C57BL/6J strain shows the greatest baseline immobility which allows for better establishment of the dose–response curve (Ripoll et al., 2003; Crowley et al., 2005). Baseline immobility in the TST also varies between strains (Bai et al., 2001; Cryan et al., 2005a; Yan et al., 2010). To avoid false negatives during antidepressant screening, it is recommendable to test the response in multiple strains. Furthermore, 70% of C57BL/6J mice, which are used as a background strain for many genetical manipulations, were found to climb their tail during TST, confounding it by learning that there is a way to escape (Mayorga and Lucki, 2001; Cryan et al., 2005a). Though other strains do not often display this behaviour, this constraint incited many researchers to continue using FST despite its shortcomings (Mayorga and Lucki, 2001). The limitation was eventually overcome with the introduction of cylindrical climbstoppers which made TST more desirable and practical than FST (Can et al., 2011b).

Forced swimming is used as a stressor during CUMS (Franklin et al., 2012). TST is thought to induce minimal stress and is more applicable in experimental paradigms involving large numbers of mice. FST and TST can have different outcomes depending on the experimental treatment and strain used (Can et al., 2011a). For example, administering imipramine to C57BL/6J strain resulted in a U-shaped dose–response curve following FST, but not TST (Bai et al., 2001). The same study found a seven-fold difference in baseline immobility between FST and TST in the NIH-Swiss strain (Bai et al., 2001). These findings might be indicative of separate underlying neuronal correlates for the two tests, which have not yet been systematically investigated. It is recommended to use both tests and compare the results to formulate conclusive findings about potential antidepressant action (Yan et al., 2010). Recent studies have found that sex plays a significant role in response to both tests. Females display higher rates of baseline immobility than males, and this difference increases following antidepressant administration (Kokras et al., 2015). Differences in auxiliary measurements, such as the head swinging frequency, were also found to differ between the sexes and females at different stages of the oestrous cycle (Kokras et al., 2015).

Despite the limitations in drug sensitivity, interstrain variation and sensitivity to environmental factors, both tests show quite high interlaboratory reproducibility (Cryan et al., 2005b; Castagné et al., 2010). FST was reported to generate false positives for some antihistamines, anticholinergics, atypical antipsychotics, and GABA agonists (Petit-Demouliere et al., 2005; Castagné et al., 2006, 2010). A major difference between the two tests is that FST is much more sensitive to sedatives—particularly 5-HT_1*A*_ agonists which decrease the immobility duration in FST but increase it in TST (Castagné et al., 2006). Previously, it was found that stimulating either 5-HT_1*A*_ or 5-HT_1*B*_ increased the immobility in FST (O’Neill, 2001). FST was also shown to induce a decrease in serotonergic activity in the hippocampus and hypothalamus of female rats while inducing an increase in serotonergic activity in the hypothalamus of male rats (Drossopoulou et al., 2004). Importantly, the level of 5-HT_1*A*_ mRNA in females’ hypothalamus was decreased, while in males’ hippocampus, it was elevated (Drossopoulou et al., 2004). SSRIs such as clomipramine, citalopram, and fluvoxamine have given false-negative results during the FST, whereas TST showed greater sensitivity (Castagné et al., 2010). In previous decades, there has not been much progress in the discovery of completely novel non-monoaminergic antidepressants, with exception of NMDA-receptor blockers, such as ketamine (Zanos and Gould, 2018; Fasipe, 2019; Fasipe et al., 2019). NMDA-receptor blockers have been reported to modulate rodent immobility in these tests (Pomierny-Chamioło et al., 2010; Holubova et al., 2014). For example, it was found that antidepressants with a serotonergic mechanism of action can be inhibited by NMDA activation, while antidepressants with a noradrenergic mechanism of action depend on AMPA activation, showcasing the possibility of heterogeneous causes of immobility in FST (Wolak et al., 2013). The findings of these studies support the involvement of serotonin and glutamate transmission in driving FST behaviours (Cryan et al., 2005b; Micale et al., 2006; Piotrowska, 2008). Additionally, glucocorticoids, CORT, acetylcholine, and dopamine signalling have been implicated in FST response (Basso et al., 2005; Addy et al., 2015; Fitzgerald et al., 2021).

The overuse of FST and TST has received an increasing amount of criticism (Nestler and Hyman, 2010; Slattery and Cryan, 2012; Molendijk and de Kloet, 2015, 2019; de Kloet and Molendijk, 2016; Trunnell, 2019; Gorman-Sandler and Hollis, 2021; Trunnell and Carvalho, 2021). The lack of development of novel antidepressants is primarily ascribed to the use of these tests (Hendrie and Pickles, 2013). Current screening using FST gave catastrophic outcomes (Trunnell and Carvalho, 2021). Out of 109 compounds tested in FST by big pharmaceutical companies, 31 were investigated in humans and only seven of these were found to have antidepressant effects. None of them has been approved for treatment. This inefficacy led to subsequent abandonment of the test (Wang et al., 2020; Trunnell and Carvalho, 2021). Historically, FST was defined as a model of depressive-like behaviour because it was sensitive to a subset of antidepressants that already had limited effectiveness (Nestler and Hyman, 2010; Hendrie and Pickles, 2013). Another issue is the assumption of equivalence between rats and mice, neither of which might be a good model organism for antidepressant screening (Hendrie and Pickles, 2013). Current antidepressant screening practices oversimplify the complexity and diversity of MDD endophenotypes. For example, behaviours such as anhedonia, sociability, and apathy are rarely reported in pharmacological studies.

Learned helplessness (LH) is another test of despair which is based on the observation that administration of repeated uncontrollable foot shocks leads to escape deficits that are reversible by a broad range of short-term antidepressant treatments (Cryan et al., 2002). A discrepancy between FST, TST, and LH is that the effects of FST and TST are acute whereas the effects of LH last for 3–5 days. Thus, some researchers differentiate resignation, as modelled by FST and TST, where there is no way to escape, and helplessness, as modelled by LH, where the animal fails to learn that the escape is possible (Castagné et al., 2009). However, it might be that the differences among FST, TST, and LH are not as substantial as it is possible that they relate to the level of induced stress which modulates the subsequent conditioned response. Moreover, it has been found that FST and TST have similar, albeit smaller, effects compared to LH on subsequent rounds of testing, which supports the idea that these tests share the same mechanisms (Cryan et al., 2002). Both FST and TST have relatively stronger predictive validity for classical antidepressants compared to LH (Cryan et al., 2002).

A major weakness of all three approaches is their face and construct validity (Bai et al., 2001; Molendijk and de Kloet, 2015; Commons et al., 2017; Nadeau et al., 2022). All three tests are based on an anthropomorphic interpretation that immobile and freezing behaviours somehow reflect the despair of the animal (Molendijk and de Kloet, 2015). Namely, the application of acute stress to naive rodents is *prima facie* quite different from human depression which occurs through combination of genetic vulnerability and chronic environmental stress (Nestler and Hyman, 2010; Pollak et al., 2010; Yan et al., 2010). Expectedly, chronic monoamine reuptake inhibitors administration failed to augment the response achieved with acute administration in FST and similarly did not have any effect on basal or stress-induced serum corticotropin concentration (Kelliher et al., 2003). Arguments have been put forward that these tests do not capture the despair, but rather, a malfunction of adaptive behaviour (Harro, 2019), functional adaptive behavioural responses (Molendijk and de Kloet, 2015, 2019; Commons et al., 2017; Gorman-Sandler and Hollis, 2021), or learning (De Pablo et al., 1989; Molendijk and de Kloet, 2015; de Kloet and Molendijk, 2016; Commons et al., 2017; Harro, 2019). Namely, immobility is thought to preserve energy and allow for heat preservation. When retested at the same temperature, only those rats tested in the 20–37°C range displayed immobility (Nadeau et al., 2022). Immobility is likely not adaptive outside of this range. The test might capture the switch between active and passive coping, in which case it would remain a valuable asset to neuropsychiatric research (Slattery and Cryan, 2012; Harro, 2019; Molendijk and de Kloet, 2019). The style of copying was found to be controlled by prefrontal cortex CRF neurons that project to GABAergic pyramidal cells (Chen et al., 2020). The reason for the true positive screening of a range of antidepressants might be that MDD and acute stress response share neural pathways, such as HPA (Kim, 2019), hypothalamus (Drossopoulou et al., 2004), hippocampus (Shishkina et al., 2010), and hindbrain serotonergic and noradrenergic nuclei (O’Leary et al., 2007). Thus, antidepressant screening might be a coincidence, which would at least partly explain false positives and negatives (Bogdanova et al., 2013).

Although not having received much attention previously, it is possible that despair is not the symptom that is being rescued, but rather psychomotor retardation (American Psychiatric Association [APA], 2013; Unal and Canbeyli, 2019; Valencia et al., 2019). This would explain the observation that antidepressant action in these tests is observed after acute administration, contrary to clinical chronic treatments (Cryan et al., 2002; Nestler and Hyman, 2010; Yan et al., 2010). It is known that psychostimulants such as amphetamine increase both swimming and general locomotor ability. Furthermore, any experimental manipulation that affects overall activity levels, such as chronic stress paradigms, can confound the conclusions of these tests by affecting locomotion. Though these alternative interpretations were primarily targetting FST use, they likely also apply to TST. However, two points should be raised regarding TST. First, TST does not incorporate hypothermia as a limiting factor. Nonetheless, the response could be interpreted in terms of energy conservation. Second, different underlying mechanisms might be driving the responses in the two tests which would not allow for such an interpolation. Either way, future studies will have to focus on elucidating the neurobiology of these tests.

The popularity of the tests is not going to shrink despite decades of research showcasing their limitations. Approximately 70% of the studies involving FST reported that the animal exhibited a “depressive-like phenotype” (Molendijk and de Kloet, 2019). Stagnation in the field, especially in the novel antidepressant discovery and the study of “despair” in animals, would benefit far more from unsuccessful attempts of new methodologies than successful reproduction of old modelling mistakes. One such attempt could be in the utilisation of these tests in modelling maladaptive acute stress responses, such as seen in autism spectrum disorder (Commons et al., 2017).

### Psychomotor changes

Psychomotor changes in patients with MDD include both psychomotor agitation and retardation (Vollmayr and Henn, 2001; American Psychiatric Association [APA], 2013; Alcantara et al., 2017). Psychomotor retardation manifests not only as an overall reduction in locomotor activities but also an inability to fulfil basic bodily needs or deliver adaptable behavioural responses (Alcantara et al., 2017). In rodents, these traits are usually assessed through the use of common tests of anxiety, such as open-field test (OFT), elevated-plus maze (EPM), and light-dark box (LDB) (Choleris et al., 2001; Walf and Frye, 2007; Ihne et al., 2012). These tests were initially developed to detect the effect of benzodiazepine-like anxiolytic drugs (Nestler and Hyman, 2010). Hence, conclusions regarding psychomotor changes based on these tests are confounded by animals’ anxiety levels. Better alternatives to these tests are monitoring home-cage activity using passive infrared sensors (Dadomo et al., 2011) or activity in the non-target trial of the social interaction test under dim red lights. Previously, wheel-running has also been used as a measure of locomotor activity. However, recent findings suggest that it has ameliorating effects on depressive-like and anxious-like phenotypes in rodents (Nishijima et al., 2013; Pang et al., 2013; Huang et al., 2017; Mul et al., 2018). It has also been suggested that FST immobility can be used to model psychomotor retardation (Unal and Canbeyli, 2019). However, it remains unclear whether a strong stressor-dictated response has strong translational value, especially given that the test is plagued with a range of confounds, as discussed in the previous section. Biological correlates of psychomotor changes have been reviewed elsewhere (Unal and Canbeyli, 2019).

### Apathy

Apathy used to be defined as a deficit in carrying out goal-oriented behaviour (Levy and Dubois, 2006; Levy, 2012), which has been amended to also include cognitive and emotional deficits that cannot be explained by psychomotor changes (Starkstein and Leentjens, 2008; Robert et al., 2009; Miller et al., 2021). There is a significant overlap between anhedonia and apathy as both involve a response to a rewarding stimulus (Husain and Roiser, 2018). Studies indicate that the underlying mechanism of apathy is disruption of reward processing which regulates motivated behaviour (Treadway and Zald, 2011; Barch et al., 2015). Important consideration should be to separate motivation from anhedonia. The question whether apathy in humans arises from decreased pleasure or the inability to execute behaviour that induces reward remains unanswered. Thus, anhedonia might represent a form of execution deficit or an altogether separate construct.

Decreased spontaneous grooming behaviour is often used to proxy apathy in rodents (Cathomas et al., 2015; Alcantara et al., 2017; Estanislau et al., 2019). Rodents spend a significant amount of time grooming themselves which they stop in conditions of elevated stress (Belzung and Griebel, 2001). Grooming is assessed through observation and scoring of coat state in the home cage (Yan et al., 2010; Planchez et al., 2019). The body is usually divided into several regions: head, neck, dorsal and ventral coats, forepaws, hind paws, genital region, and tail (Ducottet and Belzung, 2004; Yalcin et al., 2005; Smolinsky et al., 2009; Isingrini et al., 2010). Each region is given a score of 0 or 1 and the sum of all scores provides an index for the state of the score. More complex measures of grooming, such as the number of regions groomed and the probability of behaviour initiation, can be informative (Smolinsky et al., 2009). However, the finer microstructure of this complex behaviour has rarely been used as an auxiliary measure of depressive-like phenotype.

The splash test where rodents are sprayed with 10% sucrose solution which causes discomfort and induces grooming in naive animals has stronger face validity (Alcantara et al., 2017; Planchez et al., 2019). Animals exposed to stress typically exhibit a significantly lower frequency of grooming bouts, which is interpreted as a reduction in motivation to fulfil the acts of self-preservation (Smolinsky et al., 2009; Isingrini et al., 2010). However, the results are not consistent across the paradigms. For example, restraint stress increases grooming while chronic mild stress decreases it (D’Aquila et al., 2000; Willner, 2005; Yalcin et al., 2005). Both effects are reversible by antidepressant desipramine (D’Aquila et al., 2000), whereas tramadol and fluoxetine were found to increase grooming following chronic mild stress (Yalcin et al., 2005; Isingrini et al., 2010). It seems that the grooming response is dependent on the testing environment (Reis-Silva et al., 2019). The test results converge and correlate with that of SPT, FST, and TST (Pothion et al., 2004; Griebel et al., 2005; Reis-Silva et al., 2019). Besides self-grooming, apathy-like phenotypes have been assessed using reduced nest construction, maternal care, and interest in novel objects (Cathomas et al., 2015).

Less effort has been devoted to developing novel apathy models in animals. Attempts should be made outside of the self-care domain, expanding in direction of motivation, emotionality, and cognitive-performance deficits. Common approaches to studying motivated behaviour utilise a variety of effort-based decision-making tasks (Husain and Roiser, 2018). Few studies investigated the effects of chronic stress on motivation using operant-conditioning tasks, but those that did offered promising results (Dias-Ferreira et al., 2009; Dieterich et al., 2020). Besides being able to measure operant learning, these tasks allow for clear differentiation between motivation and anhedonia when coupled with the standard anhedonic measures. One limitation of the use of operant conditioning is a potential deterioration of conditioned response during chronic stress. Another understudied venue is emotionality as one of the newly included deficit responses that compose apathy. Chronic stress has been found to increase anxiety-like responses in common anxiety measures, such as EPM, OFT, and novelty-suppressed feeding, which is contrary to the expected decrease in emotionality that entails apathetic behaviour (Rosenkranz et al., 2010; Schmidt et al., 2010). Nonetheless, these tests have been used to assess emotionality, even though they are not suitable for that end (Wood et al., 2008; Lin and Sibille, 2015). Modelling of apathy would help to further reconsolidate current models and would offer a basis for exploration of systemic interactions between motivation, cognitive, and emotional maladaptive responses.

### Sociability

Reduced drive for social interaction is a hallmark of MDD (Alcantara et al., 2017). This symptom is likely a separate phenomenon from apathy, given the differences between circuits regulating sociability and motivation (Alcantara et al., 2017; Lee and Beery, 2019). Rodent social interaction tests are based on the natural tendency of rodents to interact with novel conspecifics (Gellner et al., 2021). Different brain regions, *via* different mechanisms, regulate this and the opposing behaviour of fear of novelty (Lee and Beery, 2019). The most popular sociability tests are the social interaction (SI) test and Crawley’s sociability test (CST) (Alcantara et al., 2017).

The SI test is a two-trial test conducted in an open-field arena where the rodent is left to investigate the empty arena freely in the first part of the test and is then reintroduced to the same arena with a novel conspecific social target. SI ratio score is obtained by comparing the amount of time spent in the target area with the conspecific and without it. CST is similar to SI with the difference of utilizing two testing chambers where the tested animal can either interact with the novel conspecific or familiar conspecific (Kaidanovich-Beilin et al., 2011; Alves-dos-Santos et al., 2020). CST has the advantage of being able to directly assess social memory alongside social affiliation as two different facets of sociability (Alcantara et al., 2017). Rodents tend to interact more with novel conspecifics (Yang et al., 2011). Both tests offer robust results (Golden et al., 2011; Kaidanovich-Beilin et al., 2011). Social avoidance is a robust effect of social stress, but is inducible by other paradigms, such as CUMS (Jacobson et al., 2020; Gellner et al., 2021; Morató et al., 2022). SI is commonly performed after the CSDS and is regarded as its essential behavioural test (Golden et al., 2011). Stress-susceptible mice are expected to spend less time near the conspecific and have a lower SI score than the stress-resilient mice (Krishnan et al., 2007; Biselli et al., 2021). This behaviour is reversible by antidepressants, such as tianeptine (Venzala et al., 2012). Brain regions including the hippocampus, hypothalamus, frontal cortex, and raphae nuclei are associated with social avoidance (File and Seth, 2003). Evidence suggests that mPFC pyramidal cells synchronise NAc, amygdala, and VTA at low frequencies which exerts antidepressant effects in the SI test and SPT (Kumar et al., 2013; Hultman et al., 2016). mPFC cells directly innervate GABAergic dorsal raphae nucleus (DRN) interneurons that project to neighbouring serotonergic neurons that mediate the acquisition of social avoidance following CSDS (Challis et al., 2013). Circuits driving social behaviour have been reviewed elsewhere (Lee and Beery, 2019).

There are issues with using the SI test as a definite classifier of phenotypes that emerge from CSDS. Namely, the conspecific target is usually from the aggressor CD1 strain (Golden et al., 2011). It was found that social interaction with a same-strain conspecific does not correlate with SPT (Alves-dos-Santos et al., 2020). This is contrary to earlier findings that employed social targets from the aggressor strain (Krishnan et al., 2007; Golden et al., 2011). Same-strain and aggressor social targets have opposite effects on social interaction behaviours (Venzala et al., 2012). Another study found that SI with CD1s involves a significant fear-conditioned response (Ayash et al., 2020). In another study, SI test results using same-strain target and aggressor-strain target were significantly and robustly correlated (Han et al., 2014). It is likely that using same-strain conspecific captures sociability while using aggressor-strain conspecific captures copying strategy or conditioned response. This undermines the test’s translational value as social avoidance and anhedonia are highly correlated in humans (Derntl et al., 2011). Sociability tests were originally developed for testing anxiolytic drugs where the tests include a significant anxiogenic component that varies as a function of the environment (File and Seth, 2003). It has become increasingly common to test mice in red light to decrease the anxiogenic effect of illumination (Golden et al., 2011). There is a significant interstrain variation, even when the social target is of the same strain (File and Seth, 2003; Moy et al., 2008). Future research on the value of sociability tests utilising home-cage interactions would further highlight the usefulness of sociability measures (Kim, 2019; Kim et al., 2019).

## Modelling pathogenesis

Difficulties in the development of reliable nosologic criteria lie in the current lack of comprehensive and systematic understanding of the interplay between environment and specific molecular, cellular and circuit-related pathways that lead to pathogenesis. Factors thought to trigger pathogenesis can be separated into two categories: genetic and environmental (Cross-Disorder Group of the Psychiatric Genomics Consortium, 2013; Hollander et al., 2020). Stress is considered a pathogenic feature of the highest relevance for neuropsychiatry (de Kloet et al., 2005; McEwen, 2008; Millan et al., 2012).

The diathesis-stress model posits that neither genetic predisposition nor environmental triggers alone are sufficient for disease onset, but rather that the co-occurrence of the two is required (Hollander et al., 2020). Most research studies treated these two phenomena separately, whereas only recent studies have investigated the interplay between these factors (Hollander et al., 2020). Common genetic risk factors, alongside high comorbidity, might suggest that many neuropsychiatric disorders form a continuous spectrum (Cross-Disorder Group of the Psychiatric Genomics Consortium, 2013; Anderzhanova et al., 2017).

Maintenance of homoeostasis is achieved through behavioural and physiological adaptation. Consequently, successful stress coping leads to homoeostasis reestablishment and readiness to deliver a more adaptable response in future. However, stress responses can also be maladaptive (Joëls and Baram, 2009). Severe stress is thought to interact with and exacerbate pre-existing genetic and epigenetic vulnerability (Bagot et al., 2014; McEwen, 2017). Repeated acute maladaptive responses often lead to the onset of life-long neuropsychiatric conditions (Garakani, 2006; Gelfuso et al., 2014; Gold, 2015; Gold et al., 2015). The intensity and duration of the stress interact with genetic and epigenetic predispositions, leading to the malfunction of molecular allostatic pathways (Arnsten, 2015; Chaudhury et al., 2015; McEwen, 2017; Rusconi and Battaglioli, 2018). Physiological processes of the nervous and other systems are impaired by the subsequent modifications, which will influence the success of future adaptive responses thus forming a vicious cycle (Arnsten, 2015; Chaudhury et al., 2015; Ellis et al., 2017; McEwen, 2017; Cerniauskas et al., 2019). In the last two decades the research focus has shifted from strain investigations to stress-inducing models. Currently, stress-based paradigms are thought to induce maladaptive responses that most closely resemble endophenotypes of human disorders listed in RDoC (Anderzhanova et al., 2017).

All stressors can be categorised as either physical or psychological. Lacking control over the stressor induces similar chronic behavioural changes in rodents and humans (Richter-Levin et al., 2019). Type, intensity, timing, and duration of the stressor, as well as age, sex, early-life development, and genetic susceptibility of the stressed animal, are highly variable and significant factors in determining the paradigm outcomes and have been utilised to study differential stress responses (Joëls and Baram, 2009; Lupien et al., 2009; Czéh et al., 2016; Schroeder et al., 2018). Table 2 gives an overview of the most commonly utilized stress paradigms highlighting their main strengths, weaknesses, and validity.

**TABLE 2:** Overview of the stress paradigms with respect to their strengths, weaknesses, and validity.

Paradigm	Stressor type	Human equivalent	Strengths	Weaknesses	Validity		
					Construct	Face	Predictive
Chronic unpredictable mild stress	Unpredictable mild physical stressors	Different daily life disturbances that keep on going for a longer period of time; minor issues in several spheres of life	• Induced changes persist for several weeks • Models both stress and antidepressant resilience • Particularly useful for study of sex and age factor	• Inconsistent findings • No standardised protocol • Poor reproducibility • Large time investment	Strong	Very strong	Very strong
Learned helplessness	Predictable physical stressor	Unresolvable situation or problem	• Segregation between helpless and non-helpless animals • Useful for study acute stressor effects on cognitive functions • Stress and response are quantifiable	• No association between helplessness and other behavioural measures • Inconsistent results • Effects last for up to a week • Lacks specificity for different classes of antidepressants • Highly strain-dependent	Weak	Moderate	Weak
Chronic social defeat stress	Predictable social stressor	Bullying and psychological abuse	• Naturally occurring rodent stressor • Induced changes persist for months • Continuous HPA activation • Particularly useful for study of stress resilience and vulnerability • Effects are not age-dependent	• Stressor is stronger than the ones experienced by most humans • Hardly implementable in females • Highly dependent on the social interaction test	Very strong	Very strong	Strong
Neonatal stress	Predictable or unpredictable social stressors in juvenile period	Parental negligence, childhood trauma or growing up in poverty	• Allows for the study of stress vulnerability inheritance, environmental programming and pro-resilient mechanisms	• High variation in the protocols • Often inconsistent results • Rodent’s brain developmental stage is equivalent to late foetal brain	Strong	Strong	Further research required
Chronic restraint stress	Predictable mild physical stressor	Daily unpleasant situations at work or abusive job	• Induces depressive-like phenotype in all subjected animals • Low time and space investment	• Effects subside quickly • Long time before emergence of depressive-like behaviours • HPA response quickly subsides	Moderate (Further research required)	Strong (Further research required)	Further research required

### Chronic unpredictable mild stress

Chronic unpredictable mild stress is the most widely used stress paradigm in mood disorder research (Katz et al., 1981; Willner et al., 1987; Willner, 2017; Italia et al., 2020). CUMS attempts to model unpredictability and a wide range of diverse stressors mirroring those that lead to MDD in humans. Most protocols require 1–7 weeks of daily exposure to a mild physical stressor in a randomised manner for reliable induction of depressive-like phenotype (Krishnan and Nestler, 2011; Alcantara et al., 2017). The paradigm is based on the observations that stressor controllability and expectation attenuate maladaptive behavioural and physiological changes (Pollak et al., 2010). Preferred animal models are outbred rat strains, but the paradigm has been increasingly used and validated in mice (Mineur et al., 2006). Choice of stressors should be made relative to the species used.

#### Behavioural effects

Depending on the length of the paradigm and intensity and nature of stressors used, CUMS will induce sucrose preference anhedonia, reduced self-grooming, and immobility in FST and TST in at least 40% of the mice (Willner, 2005; Mineur et al., 2006; Dalla et al., 2010; Feng et al., 2012; Overstreet, 2012). The most stable and robust readout of CUMS is anhedonia (Czéh et al., 2016; Willner, 2017). Attenuated sucrose preference and coat deterioration develop gradually (Pothion et al., 2004; Planchez et al., 2019). Anhedonia emerges a week following the paradigm’s end, supporting the necessity of prolonged continuous testing (Christiansen et al., 2016). In one study, most mice exhibited a decrease in exploratory behaviour and general locomotor activity following CUMS, but only 61% of mice exhibited anhedonia which was correlated with FST immobility (Strekalova et al., 2004). Other groups reported no effect on locomotor activity, along with a significant increase in anxiety-like behaviours, captured by OFT, LDB, and EPM (Logan et al., 2015; Cerniauskas et al., 2019). Another study showed an increase in anxiolytic behaviour (Schweizer et al., 2009). These inconsistent results may be due to variation in the type of stressors, intensity, presentation order, and strain used (Pothion et al., 2004; Mineur et al., 2006; Jung et al., 2014). Interaction between pre-existing anxiety and exploration traits predicts the behavioural outcome in a variety of tests following the CUMS (Castro et al., 2012), which is in accordance with the findings from other paradigms (Schmidt et al., 2010). CUMS was recently used to elucidate the role of projections from lateral habenula (LHb) to VTA in driving TST immobility, showcasing the construct validity of the paradigm (Bergström et al., 2007; Cerniauskas et al., 2019). Changes in aggressive and sexual behaviour have been reported, but require further quantification (Gronli et al., 2005; Couch et al., 2016; Chen et al., 2018; Wang et al., 2021). Another reported effect is the potentiation of escape deficits in the LH test (Willner, 2005). Most behavioural maladaptations following CUMS persist for several weeks (Strekalova et al., 2006; Nestler and Hyman, 2010).

#### Physiological and molecular correlates

The effects of CUMS mirror changes seen in depressed humans. Similar to other paradigms, it leads to an increase in baseline CORT levels and general activation of the HPA axis, indicating that the model is suitable for studying comorbidity between MDD and anxiety (Belzung and Lemoine, 2011). CORT levels increase due to elevated corticotropin-releasing hormone (CRH) transcription in the hypothalamus (Chen et al., 2009). Elevated CORT presumably induces the changes observed in CRH neuron density in the paraventricular nucleus (PVN) and central nucleus of the amygdala (CeA), which correlate with the depressive-like and anxiety-like phenotypes (Wang et al., 2010). Further research should confirm the role of PVN and CeA in generating the stress-susceptible behavioural phenotype following CUMS. Furthermore, local increases in mPFC CORT levels correlate with long-lasting CUMS-induced working memory impairment (Dominguez et al., 2019). CUMS also causes cognitive impairment that is associated with increased amyloid-β deposition in the hippocampus of APP/PS1 mice (Han et al., 2017). These effects correspond to the maladaptively strong response to minor stressors, as is observed in humans (Willner, 2017). CUMS is accompanied by a range of inflammatory, oxidative, apoptotic and antineurogenic responses that are reversible by antidepressant treatment (Kubera et al., 2011). Peripherally, it leads to increased levels of inflammation-inducing cytokines (Grippo et al., 2005; You et al., 2011; Remus et al., 2015). Besides systemic inflammation, an increase in hypothalamic and pituitary TNF-α and IL-1β levels suggests local inflammation of stress circuitry (Grippo et al., 2005). The increased proinflammatory response may be why MDD has significant comorbidity with systemic and peripheral organ malfunction, including its association with cardiovascular diseases (Grippo et al., 2005; Van Dam et al., 2017). CUMS also induces a range of metabolic alterations in sphingolipid, ketone, and amino-acid metabolism of the hippocampus (Han et al., 2017). It recapitulates core symptoms of metabolic syndrome: abnormal glucose levels, blood pressure, and amplified fat storage thought to be triggered by elevated proinflammatory cytokines (Dallman et al., 2000).

Stressed mice display decreased hippocampal neurogenesis and decreased neurotrophin levels (Kubera et al., 2011; Segev et al., 2014). Moreover, exposure to prenatal stress and CUMS causes decreased spine density and changes in spine morphology (Michelsen et al., 2007). CUMS also decreases serotonin neurotransmission in the forebrain and neuronal arborisation in both forebrain and hippocampus, decreased brain-derived neurotrophic factor (BDNF) and GABA signalling in the hippocampus, impaired long-term potentiation (LTP) in hippocampus-NAc projection, impaired GABA signalling in mPFC, and increased microglial activation (Holm et al., 2011; Hill et al., 2012; Ma et al., 2016; Willner, 2017). This evidence is convergent with the results from other stress paradigms, such as CSDS.

Studies using CUMS have confirmed previous predictions regarding the functional role of some molecular pathways and also led to the discovery of new candidate genes that result in mood disorders (Bergström et al., 2007; Orsetti et al., 2008; Chen et al., 2009; Surget et al., 2009; Elizalde et al., 2010; Cerniauskas et al., 2019). Circadian rhythmicity, measured by locomotor and temperature oscillations, is shifted and blunted in stress-exposed animals (Logan et al., 2015). CUMS leads to the disruption of circadian rhythms through altered expression of *Bmal1* in NAc, *Per1* in CA1, and *Per2* in CA1, CA3, dentate gyrus, and LHb (Christiansen et al., 2016). Another study found that molecular rhythms were blunted in suprachiasmatic nucleus and sharpened in NAc (Logan et al., 2015). Though CUMS offers insight into molecular pathways altered in mood disorders, high protocol variation makes it difficult to conduct follow-up studies.

#### Antidepressant discovery

CUMS is also a robust tool in pharmacological research. Induced behavioural changes are reversible by chronic administration of typical and atypical antidepressants, such as desipramine and tramadol (D’Aquila et al., 2000; Yalcin et al., 2005; Bondi et al., 2010), fluoxetine (Mutlu et al., 2012; Liu et al., 2015), citalopram (Furr et al., 2012), ketamine (Ma et al., 2013; Okine et al., 2020; Fitzgerald et al., 2021), melatonin (Haridas et al., 2013), and AMPA receptor potentiator (Farley et al., 2010). Rescue occurs after 2–5 weeks of treatment, which corresponds to antidepressant response in humans (Yan et al., 2010). Antidepressant-induced decrease in hippocampus cytogenesis, which correlates with symptoms amelioration, as observed in clinical studiesm has also been confirmed in CUMS (Jayatissa et al., 2006). Additionally, models of antidepressant resilience have been developed using adapted versions of CUMS and a high-fat diet (Isingrini et al., 2010; Finger et al., 2011).

#### Strengths and weaknesses

A specific advantage of the paradigm is that it segregates two distinct populations: antidepressant-sensitive and antidepressant-resistant (Jayatissa et al., 2006). Typically, around 50% of animals will not be rescued by antidepressant treatment (Czéh et al., 2016). Robust and converging behavioural output can be tracked to underlying neuronal correlates using transcriptional, physiological, and synaptic levels of analysis (Cerniauskas et al., 2019). Mortality in this paradigm is much lower than in other paradigms, such as CSDS. Furthermore, CUMS allows for robust studies of stress-induced molecular perturbations (Logan et al., 2015; Christiansen et al., 2016). Overall, the paradigm is thought to have a high face, construct, and predictive validity (Nestler and Hyman, 2010; Yan et al., 2010). However, the paradigm is time-consuming and labour-intensive (Yan et al., 2010; Alcantara et al., 2017). Moreover, questions regarding the reliability of the model have been raised (Cryan and Mombereau, 2004; Willner, 2005, 2017). Poor reproducibility stems from protocol variation (Krishnan and Nestler, 2011; Willner, 2017). CUMS remains a particularly useful tool for studying sex and age factors due to the mildness of the stressors and length of the paradigm, something that is particularly difficult to model in other paradigms. Significant differences in neuroanatomy, physiology, and molecular mechanisms of stress between the two sexes and various age groups, which require further research, have already been identified (Bielajew et al., 2003; LaPlant et al., 2009; Franceschelli et al., 2014; Pfau and Russo, 2015; Heck and Handa, 2019).

### Learned helplessness

Learned helplessness attempts to model reduced overall capability and effectiveness, which are intimately associated with apathy and behavioural despair (Alcantara et al., 2017). The paradigm consists of placing a rodent in a chamber with an electrified floor from which it cannot escape and where it is repeatedly exposed to unpredictable foot shocks (Planchez et al., 2019). After one or several rounds of conditioning, the animal is given an opportunity to stop or escape the shocks (Vollmayr and Gass, 2013). Multiple conditioning sessions will result in a diminished motivation to escape (helplessness), even when escape is possible, as measured by latency to press the lever or move to another chamber (Alcantara et al., 2017). This behaviour is viewed as a coping deficit reminiscent of mood disorders (Vollmayr and Gass, 2013). The test was originally developed for rats, which exhibit robust helpless phenotype up to a week after the acquisition phase (Seligman et al., 1975). The response in mice lasts for 2–3 days (Krishnan and Nestler, 2011). Rats’ escape latency is usually assessed using a lever press, whereas mice are commonly tested in shuttle boxes (Vollmayr and Henn, 2001; Chourbaji et al., 2005).

#### Behavioural effects

The paradigm segregates helpless (susceptible) and non-helpless (resilient) animals (Kim Y. et al., 2016, Kim et al., 2017). The paradigm can be used to investigate the effects of lack of control, as well as different levels of control in the experimental animals (Alcantara et al., 2017). The response to the paradigm is highly variable where 10–80% will fail to acquire the helpless phenotype (Krishnan and Nestler, 2011). The model is useful for investigating the effects of inescapable acute stress on cognitive processes (Song et al., 2006; Yan et al., 2010). There is no strong association between helplessness and anhedonia, which significantly weakens the validity of the model (Kim et al., 2017; Song and Vilares, 2021). The absence of a consistent anhedonic response is unexpected given the decreased dopamine levels in VTA (Uliana et al., 2020). Interestingly, mice that underwent LH, irrespective of escape deficits, displayed increased anhedonic response and anxiety, as measured by SPT and OFT (Kim et al., 2017). Results regarding correlation of helpless phenotype and immobility remain inconclusive (Su et al., 2016; Kim et al., 2017) mice selectively bred for helplessness develop anhedonia and other depressive-like traits mediated through an increase of βCaMKII in the LHb following exposure to acute LH stress (Vollmayr et al., 2004; Li et al., 2013; Nam et al., 2014; Anpilov et al., 2020). Furthermore, “helpless” rodents display reduced locomotion and sexual behaviours (Dess et al., 1988).

#### Physiological and molecular correlates

Learned helplessness causes significant increases in CRH, CRF, and CORT levels, through HPA axis dysregulation (Greenberg et al., 1989; Song et al., 2006). LH also significantly alters REM sleep and body weight (Dess et al., 1988; Adrien et al., 1991). Decreased levels of IL-2 in a variety of regions implicated in MDD were reported following LH (Lee et al., 2008). IL-1β was found to mediate an increase in escape latency (Bonaccorso et al., 2003). These findings led to a fruitful investigation of immunological causes and effects of LH (Kubera et al., 2011). Neural effects include decreased hippocampal neurogenesis (Malberg and Duman, 2003), loss of spine synapses in the hippocampus (Nestler et al., 2002; Cryan and Mombereau, 2004), and overall decreased levels of expression of synaptic and cytosolic proteins (Reinés et al., 2008). However, helpless behaviour preceded a maximal decrease in hippocampal neurogenesis which was observable in all mice that underwent LH (Vollmayr et al., 2003; Henn et al., 2004). Inescapable shock is followed by strong activation of DRN serotonergic neurons which modulate downstream targets, such as the NAc, amygdala, and dorsal periaqueductal grey (Maier and Seligman, 2016). Helpless rats exhibit elevated dopamine levels in the hippocampus and PFC and decreased dopamine levels in the amygdala relative to stress-naive and non-helpless rats (Muneoka et al., 2020). Non-helplessness was associated with decreased noradrenaline in the amygdala and increased serotonin levels in the NAc, further confirming the importance of the NAc-amygdala projection for the induction of helplessness (Muneoka et al., 2020). LH leads to increased excitability of LHb cells projecting to the VTA in helpless mice (Li et al., 2011). This is consistent with findings from other paradigms (Browne et al., 2018; Gold and Kadriu, 2019). The increased LHb activity and decreased dopamine signalling are indicative of coping deficits in the helpless response (Shumake et al., 2010; Ilango et al., 2012). Impairment of portions of PFC in adolescence led to the display of helpless behaviour in adulthood (Uliana et al., 2020).

Learned helplessness is associated with a decrease in BDNF and CREB levels in the hippocampus (Song et al., 2006; Shirayama et al., 2015). BDNF administration can reverse LH-induced deficits (Kubera et al., 2011). Midbrain BDNF injections confer the non-helpless phenotype, whereas injections into the dentate gyrus prevent stress-induced hippocampal BDNF reduction (Siuciak et al., 1997; Greenwood et al., 2007). BDNF levels are decreased due to the binding of silencing HDAC5 to the BDNF promotor (Su et al., 2016). Helpless and non-helpless exhibit the expression of distinct sets of genes (Kohen et al., 2005; Berton et al., 2007). For example, helpless mice exhibit higher night-time expression of core circadian clock gene cryptochrome (*CRY*) in NAc, which was associated with decreased activation of D1-expressing medium spiny neurons (MSN) (Porcu et al., 2020). Knockdown of *Cry* in these neurons increased NAc neuronal activation at night and safeguarded against LH-induced behavioural deficits (Porcu et al., 2020). Overall, the greatest changes in expression profile were observed in non-helpless mice, whereas escape-deficient mice have a similar expression pattern as controls (Kohen et al., 2005). Interestingly, elevated activation of ΔFosB in the midbrain’s periaqueductal grey promotes non-helplessness partly through transcriptional repression of *substance P* (Berton et al., 2007). These findings in LH suggest that resilience is an active process that mobilises a range of pathways to buffer against stress. A similar active coping response is seen in mice resilient to CSDS, which suggests shared underlying mechanisms (Russo et al., 2012; Friedman et al., 2016; Fisher et al., 2018).

#### Antidepressant discovery

Learned helplessness has been used for generating a variety of genetic animal models through selective breeding and antidepressant testing where the responses largely correspond to FST (Henn and Vollmayr, 2005). Unlike acute drug administration in FST and TST, only repeated subchronic antidepressant treatment over 3–5 days will rescue the animals from the induced helplessness (Ramaker and Dulawa, 2017; Planchez et al., 2019). For example, a week-long fluoxetine treatment led to the reversal of both the escape deficits and attenuated hippocampal neurogenesis (Malberg and Duman, 2003). Then, 3-week-long fluoxetine treatment reversed the changes in cytosolic and synaptic proteins (Reinés et al., 2008). TCAs, monoamine oxidase inhibitors, SSRIs, and other classical drugs were found to be effective in reversing behavioural, physiological, and molecular changes (Weiss et al., 1981; Song et al., 2006; Reinés et al., 2008). However, LH lacks specificity and does not recognise different classes of antidepressants (Ramaker and Dulawa, 2017). Importantly, anxiolytics and stimulants cannot reverse helplessness (Pollak et al., 2010). Nonetheless, some substances that do not have any effect on humans were screened as having antidepressant action in the LH (Ramaker and Dulawa, 2017). The antidepressant action of some drugs lasts for only a day which implies the inability of LH to predict antidepressant onset (Ramaker and Dulawa, 2017).

#### Strengths and weaknesses

Humans do exhibit learned helplessness under the conditions of unpredictable and uncontrollable stress (Hiroto and Seligman, 1975; Miller and Seligman, 1975; Abramson et al., 1978). This response is not attributable to the effects of stress itself, but rather to perceived mutual independence of response and outcome (Song and Vilares, 2021). However, MDD in humans is not primarily generated through this kind of learning, even if cognition and learning that stressors are uncontrollable and unpredictable play a critical role during pathogenesis. Apathy and behavioural despair cannot be assigned equivalency with helpless behaviour likely due to the acute and relatively intense nature of the shock stressor and possibility of escape. LH is, however, advantageous because it allows for simple quantification of some kind of motivational state, learning response, and amount of stress that the animal receives. There are major disadvantages to using this model as well. First, escape deficiency poorly correlates with other behavioural measures (Pollak et al., 2010; Kim et al., 2017). A significant confounding factor is that the most widely used protocols do not dissociate fear conditioning and learned helplessness. A recent protocol dissociating fear conditioning from helplessness has been developed to model *trans*-situational of helpless response, but this protocol has also failed in producing robust differences in behavioural tests (Landgraf et al., 2015; Kim et al., 2017). The strong sensitivity of LH to central and peripheral pain processing might partly explain the high variation, along with protocol and strain variation (Cryan and Mombereau, 2004; Kubera et al., 2011). There is a large discrepancy in popular protocols (Mitchell and Redfern, 2005). Second, most of the induced behavioural and physiological changes do not persist more than a couple of days following the termination of the uncontrollable shock, which weakens the face validity of the model (Weiss et al., 1981; Cryan et al., 2002). However, some studies report considerably longer retention periods of 10–14 days (Kubera et al., 2011). Finally, a considerable number of mouse strains have endogenous escape deficiencies completely independent of the controllability of the stressor (Hitzemann, 2000). For example, Kyoto and Charles River Holtzman rat lines, and Balb/cByJ and C3H/HeJ mouse lines are highly susceptible to LH (Anisman et al., 2001; Henn and Vollmayr, 2005). Commonly used mouse lines that display robust behaviour in other paradigms, such as C57BL/6J and DBA/J, have only a moderate response in LH (Anisman et al., 2001). Interestingly, it has been found that female rodents do not exhibit helplessness and this outcome is not attributable to hormonal differences (Dalla et al., 2008). These differences might rather be explained by higher ambulatory activity in the females or greater indices of novelty reactivity (Padilla et al., 2009). This represents a potential future research venue.

### Chronic social defeat stress

Chronic social defeat stress is a homotypic stress model that induces core symptoms of MDD and PTSD in rodents (Krishnan et al., 2007). It consists of forcing the experimental rodent to intrude into the home cage of a more aggressive resident which will attack and try to dominate the intruder (Alcantara et al., 2017). After a brief direct physical contact, the intruder is housed in the same cage as the resident for the remainder of the day, kept separated by a transparent divider that limits physical and allows for sensory contact (Alcantara et al., 2017). The procedure is repeated consecutively for 10–21 days during which the intruder is daily introduced to an unfamiliar resident. Two subpopulations of stressed mice emerge following CSDS: stress-susceptible and stress-resilient (Golden et al., 2011). Resilient mice mostly display identical behaviour to that of stress-naïve mice. However, they exhibit a range of neuroanatomical and physiological differences. Differences in molecular biomarkers indicate that resilience is an active mechanism and not a passive property (Bagot et al., 2015; Anacker et al., 2018). Another two related paradigms exist: subthreshold CSDS (SDSS) and vicarious social defeat stress (VSDS). SDSS involves shorter defeat sessions, sensory interaction, and overall duration of the paradigm and is useful for studying transient effects of social defeat (Chaudhury et al., 2013; Biselli et al., 2021).

#### Behavioural effects

The hallmark and most robust behavioural outcome of CSDS is social avoidance as measured by the SI (Berton et al., 2006; Krishnan et al., 2007; Golden et al., 2011). Mice that display social avoidance are deemed as susceptible. Susceptible and resilient mice have different behavioural, physiological, and molecular profiles (Krishnan et al., 2007). Susceptible mice display reduced exploration, increased immobility, and anhedonia (Avgustinovich et al., 2005; Krishnan et al., 2007; Iñiguez et al., 2014). Equivalent outcomes have been reported in rats (Rygula et al., 2005). Learning, memory, and cognition are also affected (Von Frijtag et al., 2000; Yu et al., 2011). Both susceptible and resilient mice show an increase in anxiety-like behaviour in the EPM, LDB, and fear conditioning paradigms (Golden et al., 2011; Venzala et al., 2012; Bosch-Bouju et al., 2016). One other study found that resilient mice display enhanced fear expression and poor extinction, but not anxiety-like behaviour, relative to controls and stress-susceptible mice (Meduri et al., 2013). Hence, behavioural findings are often inconsistent.

#### Physiological and molecular correlates

One key difference between CSDS and other paradigms is that it allows for systematic continuous activation of the HPA axis (Covington and Miczek, 2005; Schmidt et al., 2010). CSDS induces a range of metabolic changes through hormonal dysregulation. Stressed mice have lower levels of adipose tissue and serum leptin that are mediated through β_3_-adrenergic signalling, which is indicative of insulin and leptin resistance (Chuang et al., 2010a; Nestler and Hyman, 2010). This pathway was found to have ameliorating effects on behavioural traits (Chuang et al., 2010b). Susceptible mice display decreased richness and diversity of the gut microbiome and altered immunoregulatory responses (Bharwani et al., 2016). Given the region-specific changes in BDNF and that increased BDNF increases inhibition of inhibitor κ kinase and associated cytokine release, the main mechanism driving inflammation remains yet to be uncovered (Christoffel et al., 2011b; Koo et al., 2019). The circadian rhythms of temperature locomotion and sleep are also disrupted following CSDS (Tornatzky and Miczek, 1993; Meerlo et al., 2002; Wells et al., 2017; Radwan et al., 2021). VTA and basolateral amygdala showed significant downregulation of *Per2* circadian expression in defeated mice, whereas PFC and NAc showed significant upregulation (Wells et al., 2017).

Many biomarkers of human depression are equivalently disrupted in susceptible mice following CSDS. Susceptible mice exhibited higher rates of neurogenesis in hippocampal dentate gyrus relative to stress-naive and resilient mice 4 weeks following CSDS (Lagace et al., 2010). CSDS transiently attenuates hippocampal neurogenesis of stressed mice, irrespective of social avoidance (Lagace et al., 2010; Tse et al., 2014). Dendritic remodelling of hippocampal neurons induces the changes in hippocampal long-term depression and LTD, and these changes, along with increased neurogenesis, are thought to allow for a dynamic range of adaptive neuroplasticity (Buwalda et al., 2005). Resilience is mediated through the activity of ΔFosB in glutamatergic ventral hippocampal neurons (Eagle et al., 2020). Interestingly, suppression of these neurons is required for goal-oriented behaviour, which is dysregulated in a subset of stressed mice (Yoshida et al., 2021).

Several different regions regulate response to CSDS. Most of these subcircuits involve VTA. For instance, it has previously been found that susceptible but not resilient mice exhibit high-frequency phasic firing in dopaminergic VTA neurons projecting to NAc and that inhibition of dopaminergic VTA neurons projecting to mPFC induces susceptible phenotype (Chaudhury et al., 2013). Increasing even further the hyperactivity of VTA dopaminergic neurons to match that of resilient mice reverses susceptible phenotype (Friedman et al., 2014). Another study found that decreased firing of serotonergic projection from VTA to DRN induces the susceptible phenotype (Zou et al., 2020). Taken together, these three projections likely form the crux of the circuit that drives depressive-like behaviour. VTA-induced susceptibility to CSDS was found to be caused by decreased firing in inhibitory noradrenergic projection from locus coeruleus through the action of α_1_ and β_3_ adrenergic receptors (Isingrini et al., 2016; Zhang H. et al., 2019). Interestingly, elevated dopaminergic VTA firing is associated with activated AKT, which is necessary and sufficient to induce increased immobility and reduced sucrose preference (Krishnan et al., 2008).

CSDS increases stubby spine densities in the NAc irrespective of age, through an increase of IκB kinase (Christoffel et al., 2011a; Warren et al., 2014). This pathway is necessary and sufficient for the induction of social avoidance (Christoffel et al., 2011a). Moreover, unique patterns of synaptic reorganisation occur in susceptible, but not resilient mice (Christoffel et al., 2011a). Another correlate of the susceptible phenotype is decreased levels of histone deacetylase 2 in NAc, which leads to an increase in acetylated histone H3 and persistent changes in the transcriptomic and epigenetic landscape (Covington et al., 2009; Wilkinson et al., 2009). Induction of transcription factor ΔFoSB in NAc induces transcription of the GluR2 AMPA receptor subunit, which decreases the excitability of NAc neurons by glutamate (Vialou et al., 2010). This pathway is both necessary and sufficient for resilience. One key gene in NAc that modulates susceptible and anxious behavioural response to CSDS in NAc is *SIRT1* (Kim H. D. et al., 2016). Another important factor in NAc is VTA-derived BDNF, which is required for the development of avoidant phenotype and whose knockdown prevents most CSDS-induced effects (Berton et al., 2006). Interestingly, BDNF is decreased in VTA of susceptible rats displaying chronic brain reward deficits (Der-Avakian et al., 2014). Future studies will have to uncover the relationship between NAc and VTA BDNF levels during and following defeat. The importance of cell-type microdissection is demonstrated by the observation that histone acetylation in NAc’s D2-MSN and histone methylation in D1-MSN neurons promote the stress-susceptible phenotype whereas the opposite processes promote the stress-resilient phenotype (Hamilton et al., 2018).

mPFC also plays an important role as its dysregulation, induced by CSDS, leads to increased synchronisation of VTA and amygdala activity and induction of susceptibility (Hultman et al., 2016). mPFC induces the proinflammatory cytokine release and hypercortisolaemia following CSDS (Hinwood et al., 2012; Reader et al., 2015). Neuronal and microglial activation co-occurs in several anxiety and fear-related brain regions (Reader et al., 2015), potentially leading to LTP in these regions which may explain the high anxiety–depression comorbidity (Hinwood et al., 2012). Susceptible mice show stronger fear responses in auditory fear conditioning paradigms mediated through higher BDNF levels in the basolateral amygdala, indicating the importance of this pathway for comorbidity between anxiety and MDD (Chou et al., 2014).

#### Stress vulnerability

This model is very useful for investigating vulnerability to stress. Besides different firing patterns, the volume size of particular regions of interest, such as VTA, LHb, and hypothalamus, is positively correlated with social avoidance following CSDS (Anacker et al., 2016). Socially dominant and subordinate mice differ in their behaviour (Bartolomucci et al., 2004). These categories are roughly equivalent to resilient and susceptible phenotypes, respectively (Larrieu et al., 2017). The significance of this finding is that difference between susceptible and resilient mice in terms of behavioural coping style precedes physiological separation that occurs around the 9th day of 10–21 days of defeat. Behavioural characterisation of intruder mice’ copying styles using deep neural networks would provide further insight into the emergence of these two phenotypes. Dominant mice, which were previously found to be more vulnerable to the effects of social stress, display fragmented sleep architecture (Larrieu et al., 2017; Karamihalev et al., 2019). Sleep fragmentation was also found to occur through higher rates of NREM-to-Wake switching in stress-vulnerable and susceptible mice (Radwan et al., 2021). Behavioural and physiological studies imply that increased stress reactivity, possibly induced by differences in CRH, is a major predictive factor of stress vulnerability (Schmidt et al., 2010; Wood et al., 2010; Milic, 2021). Interestingly, low-novelty-seeking rats proved to be resilient to CSDS and exhibited a post-defeat increase in hippocampal BDNF (Duclot and Kabbaj, 2013). One systemic study found that smaller hippocampal volume, anxious traits, and elevated systemic IL-6 were reliable predictors of vulnerability to stress (Nasca et al., 2019). An important remark is that anatomical and physiological features in susceptible mice can predate the stress and present predictors, rather than effects, of CSDS outcome. The vulnerability-encoding network, which is biologically distinct from the networks encoding post-defeat maladaptations, originates in the PFC and ventral striatum is transmitted through the amygdala and VTA and converges at the ventral hippocampus (Hultman et al., 2018).

#### Antidepressant discovery

Resilient and antidepressant-treated mice exhibit similar patterns of transcriptional activation (Vialou et al., 2010) and chromatin remodelling (Covington et al., 2009; Wilkinson et al., 2009). This indicates that resilient mice utilise a sort of endogenous antidepressant-like response (Krishnan and Nestler, 2011). Effects induced by CSDS can last up to 6 months and are reversible by chronic, but not acute, antidepressant treatment (Von Frijtag et al., 2000; Berton et al., 2006; Tsankova et al., 2006; Rygula et al., 2008; Alcantara et al., 2017; Becker et al., 2021). Some of the effective antidepressants include imipramine (Wilkinson et al., 2009), citalopram (Rygula et al., 2006b), fluoxetine (Rygula et al., 2006a; Vialou et al., 2010), venlafaxine (Venzala et al., 2012), escitalopram (Yoshida et al., 2021), and high doses of ketamine which induce only transient avoidance attenuation (Donahue et al., 2014). Fluoxetine’s chronic effects are exerted through decreased binding of ΔFoSB to CaMKIIα promotor (Robison et al., 2014). Increased expression of CaMKIIα has been associated with the susceptible phenotype in several stress-related nuclei (Robison, 2014). Sleep deprivation and voluntary wheel-running likewise attenuate and prevent many effects of CSDS (Von Frijtag et al., 2000; Zhang J. et al., 2019). Chronic effects of CSDS are observable only in post-defeat single-housed, but not socially housed, rodents (Von Frijtag et al., 2000; Buwalda et al., 2005). Anxiolytic benzodiazepines cannot reverse the effects of CSDS, which indicates that the simultaneous increase in anxious-like behaviour of both susceptible and resilient populations is not the driver of other biological changes (Berton et al., 2006). Thus, the paradigm separates depressive-like and anxious-like phenotypes.

#### Strengths and weaknesses

Overall, CSDS displays relatively high construct, face, and predictive validity, at least for monoaminergic drugs (Tsankova et al., 2006; Nestler and Hyman, 2010; Golden et al., 2011). This model has particular value since rodents encounter social stress in their natural environment. Other paradigms, such as CUMS and LH, seem artificial. Furthermore, the majority of stressors that trigger depression in humans are of social nature (Brown and Prudo, 1981; Björkqvist, 2001; Krishnan and Nestler, 2011). However, the stress in CSDS is more severe than one commonly experienced by humans (Nestler and Hyman, 2010). CSDS has become very popular in mice research because it is more straightforward than CUMS and, unlike in rats, it does not require the presence of a female to induce male-to-male aggression (Alcantara et al., 2017). CSDS, unlike most other stress paradigms, produces a robust, reliable, and persistent phenotype, regardless of the age of the animals (Iñiguez et al., 2014). CSDS can be used to study resilience and its underlying mechanisms, which gives it significant value over other paradigms (Nestler and Hyman, 2010; Alcantara et al., 2017). Approximately 40% of mice that undergo CSDS will display a resilient phenotype (Golden et al., 2011). This is equivalent to the human condition where only a subpopulation is susceptible to stress, giving significant face validity to the model (Alcantara et al., 2017).

A major disadvantage of this model is that it is not applicable to females (Yan et al., 2010). Recently, chemogenetic activation of the ventromedial nucleus of the hypothalamus was used to induce aggression toward the female intruder, yielding a similar susceptible phenotype following CSDS (Takahashi et al., 2017). CSDS is thought to model both MDD and PTSD which is important given the high comorbidity between the two disorders (Nestler and Hyman, 2010; Pollak et al., 2010; Flory and Yehuda, 2015). A large emphasis on social avoidance and MDD modelling suppressed important considerations regarding modelling PTSD using CSDS. In either case, additional research will be required to uncover the validity of using CSDS as a model for PTSD. The use of SI as the main behavioural classifier does have shortcomings. Given the complexity and multifacetedness of MDD that CSDS attempts to model, the claim that one type of behaviour, which is not even dysregulated in all depressed humans, is the best classifying tool in rodents is simply unfounded. Indeed, CSDS induces many behavioural phenotypes, which are shared by stressed mice and depressed patients, but which are completely unrelated to social avoidance, such as goal-oriented behaviour (Yoshida et al., 2021). Reliable classifiers of different behavioural endophenotypes can be built independent of social interaction and allow for the discovery of important and robust biological correlates (Bosch-Bouju et al., 2016). Hopefully, future studies will decrease the dependence of CSDS phenotyping on the SI test.

### Vicarious social defeat stress

The underexplored VSDS is a paradigm that attempts to induce depressive-like symptoms in mice by having them witness another mouse’s defeat. Current protocols allow for the simultaneous running of CSDS and VSDS (Warren et al., 2014). Their effects on some pathways are similar, but significant differences exist. For example, whereas CSDS increases spine density in NAc of both adolescent and adult mice, VSDS does so only for the adolescent mice (Warren et al., 2014). One advantage of VSDS over CSDS is the successful recapitulation of depressive-like behaviours in females, including attenuated sucrose preference, increased immobility in TST, and physiological abnormalities, such as increased CORT and decreased body weight (Iñiguez et al., 2018). The paradigm was reviewed elsewhere in-depth (Sial et al., 2016; Warren et al., 2020).

### Prenatal and neonatal stress

An important factor dictating the progression and severity of MDD and comorbidity rates with other disorders is childhood trauma and adversity (Heim et al., 2008; Zisook et al., 2022). Questions regarding the effect of early-life experiences have received an increasing amount of attention, particularly in the context of predicting stress vulnerability or inducing resilience. Prenatal stressors applied in early and late gestation periods are less commonly used in stress research even though they most often produce robust effects in later life (Weinstock, 2008). Most early-life stress (ELS) paradigms are delivered within the first 3 postnatal weeks. Neonatal stressors most often involve social bond disruption, as in the case of maternal separation and deprivation, but other stressors, such as foot shock and restraint stress, also produce depressive-like effects (Alcantara et al., 2017).

#### Prenatal stress

Prenatal stress paradigms involve the daily application of mild stressors to the pregnant dam throughout the gestation period. These stressors include circadian stressors, restraint stress, sleep deprivation, mild shocks, crowding, and forced swimming (Bock et al., 2011; Zheng et al., 2016; Baratta et al., 2020). The elevated stress hormones, CRH, catecholamines, and glucocorticoids cross the placenta and affect the developing foetal brain (Coussons-Read, 2013). Later into adulthood, these mice exhibit anhedonic and anxiety-like behaviour, alongside increased immobility in FST and TST (Akatsu et al., 2015; Jia et al., 2015; Zheng et al., 2016; Soares-Cunha et al., 2018). Learning and spatial memory are also impaired (Wu et al., 2007; Maur et al., 2012). Besides these behavioural changes, the offspring exhibits increased basal CORT levels and hyperactive HPA responsiveness to acute stress (Morley-Fletcher et al., 2003; Soares-Cunha et al., 2018). In these rodents, glucocorticoid receptors and CRF expression are upregulated, whereas hippocampal BDNF is downregulated (Mueller and Bale, 2008; Jia et al., 2015; Zheng et al., 2016). Morphological and connectivity changes in hippocampal and PFC dendritic arborisation are commonly reported (Murmu et al., 2006; Michelsen et al., 2007; Bock et al., 2011; Mychasiuk et al., 2012) prenatal stress induces long-term and transmittable epigenetic changes which differ between the sexes (Patin et al., 2005; Zuena et al., 2008; Provençal and Binder, 2015). However, unlike with postnatal stressors, prenatal most of these periods are observable throughout the early postnatal, adolescent, and adulthood changes. More extensive reviews on prenatal stress have been published (Boersma and Tamashiro, 2015; Weinstock, 2017).

#### Neonatal stress

Early-life maternal separation (ELMS) consists of the daily separation of the pups from their mother for 15–240 min, most commonly between postnatal days 2 and 20 (Andersen, 2015; Marco et al., 2015). Some other models of neonatal stress are poor parental care (Lo Iacono et al., 2021), repeated cross-fostering (Luchetti et al., 2015), and denied expected reward (Diamantopoulou et al., 2018). Several studies also introduced the changes in rearing conditions through limited nest bedding (Raineki et al., 2012; Arp et al., 2016), changes in the bedding type (Coccurello et al., 2014; Arp et al., 2016; Goodwill et al., 2019), and communal nursing paradigms in mice and rats (Branchi and Alleva, 2006; Macrì et al., 2010). Stressful rearing conditions likely exert their effect directly and independently of maternal care, as hypothesised by the modern maternal moderation hypothesis (Macrì and Würbel, 2006).

##### Behavioural changes

Pups that underwent neonatal stress grow to become more submissive and adopt a more passive stress coping style (Gardner et al., 2005). Motivation deficits and the emergence of anhedonia are also commonly reported in sucrose-based and other reward tasks (Leventopoulos et al., 2009; Coccurello et al., 2014; Goodwill et al., 2019; Kangas et al., 2022). ELMS induces a subthreshold effect in ICSS that emerges only following chronic adulthood stress (Matthews and Robbins, 2003; Der-Avakian and Markou, 2010). However, the results are not consistent as another study found that the effect was specific only to females (Michaels et al., 2007). Adult rodents that underwent ELMS exhibit elevated anxiety-like behaviour most consistently in EPM, but also in OFT (Romeo et al., 2003; Lee et al., 2007; Ishikawa et al., 2015; Goodwill et al., 2019). Anxiety-like behaviour due to ELMS is not observable in adolescence (Marco et al., 2015). Elevated anxiety is likely partly due to the earlier emergence of fear and extinction learning that is typically observed in adulthood (Callaghan and Richardson, 2011; Arp et al., 2016). ELMS impairs recognition memory and impacts cortical functioning (Janetsian-Fritz et al., 2018). Males show larger deficits in hippocampus-dependent cognitive functions, whereas females display less prominent cognitive dysfunction in only a subset of the cognitive tasks (Naninck et al., 2015). Early-life restraint stress and foot shocks also impair spatial learning in adult male mice (Weinstock, 2008). Many of these behavioural changes are highly strain, sex-, and age-dependent (Millstein and Holmes, 2007; Kundakovic et al., 2013; Goodwill et al., 2019). For example, working memory is specifically impaired in male rodents (Frankola et al., 2010; Brenhouse and Andersen, 2011). Even though working memory impairment is predicted by growing up in a poor household, sex is not a significant factor (Evans and Schamberg, 2009). Contrastingly, ELMS males exhibit higher deficiency in escape attempts in LH, whereas females show a higher deficiency in escape latency following first exposure (Leussis et al., 2012). ELMS induces increased immobility in FST and decreased overall locomotion in Sprague-Dawley, irrespective of sex (Lee et al., 2007), and only female Long-Evans rats (Mourlon et al., 2010). Immobility duration does not differ between the sexes in early adolescence, but in adulthood, male mice display shorter, immobility duration than females (Goodwill et al., 2019).

##### Physiological and molecular correlates

Early-life adversity permanently changes the endocrine response (Plotsky et al., 2005). Neonatal stress paradigms result in the strengthening of HPA axis responsiveness (Pryce et al., 2005; Enthoven et al., 2008; Nishi et al., 2013). Consistently, CORT levels are elevated following an acute-stress exposure in adult mice that underwent neonatal stress (Plotsky et al., 2005; Kember et al., 2012). ELS also affects neurotrophin levels, most notably in the hippocampus (Marais et al., 2008). This perturbation is one of the factors that predispose the individual to an elevated stress vulnerability in adulthood (Heim et al., 2008). In rodents, HPA axis activity and stress responses remain low during early postnatal life but exacerbate later in adulthood (Levine, 2005). Relatedly, basal adrenocorticotropic hormone levels are higher in adult rats that underwent ELMS (Newport et al., 2002; Daniels et al., 2004). A more complete recent review of ELS-induced changes in HPA and related hormones has been published (van Bodegom et al., 2017). Another robust effect of neonatal stressors in rodents is sleep disturbances which correspond to increased insomnia rates in individuals that experienced childhood traumas (Tiba et al., 2004; Palagini et al., 2015). Only long maternal separation, but not brief maternal separation, induces abnormal EEG and sleep patterns in adulthood (Mrdalj et al., 2013). Finally, different neonatal paradigms have varying effects on visceral and somatic pain hypersensitivity in male and female rats, which might play a role in subsequent susceptibility to defeat paradigms in adulthood (Prusator and Greenwood-Van Meerveld, 2016).

The majority of the changes in the brain following ELS have been investigated in the PFC-hippocampal-amygdala circuit (Smith and Pollak, 2020). Early-life restraint stress and foot shocks decrease dendritic spine densities in the hippocampus, which induces impaired spatial learning (Weinstock, 2008). Conversely, ELMS augments the number of CRH neurons in the hippocampus and activation of CRF_1_ receptors in cortical pyramidal neurons, causing poor memory performance in the Morris water maze (Ivy et al., 2010). Children who experienced maternal deprivation exhibit earlier emergence of adult-like amygdala-PFC connectivity, confirming the *trans*-species validity of results obtained in rodents (Gee et al., 2013; Ishikawa et al., 2015; Johnson et al., 2018). Moreover, as in humans, rodent amygdala and hippocampal volume changes predict greater behavioural changes in adulthood (Rao et al., 2010; Hanson et al., 2015). ELMS accelerated the development of hippocampal-dependent context-conditioned fear learning in mice which is in line with exacerbated brain maturation in traumatized children (Bath et al., 2016; Ruiz et al., 2018). Both hippocampus and PFC exhibit a marked decrease in the number of neurons, amounts of vesicle integral glycoprotein synaptophysin, markers of synaptic plasticity, and dendritic arborisation (Oomen et al., 2010; Farrell et al., 2016; Cui et al., 2020). Furthermore, several hippocampal and PFC subregions displayed the signs of long-term neuroinflammation – decreased astroglial and increased microglial populations (Banqueri et al., 2019). Many plasticity and synapse-related changes observed in the hippocampus are likely a direct consequence of alterations in microglial numbers, gene expression, and phagocytic activity which persist well into the juvenile period (Johnson and Kaffman, 2018). Following neonatal stress, the LHb, another nucleus that modulates mood, exhibits increased excitability and burst firing which is reverseable by a single ketamine injection (Shepard et al., 2018). Future studies should aim to address the literature gap regarding the role of other stress-related brain nuclei and their electrophysiological states in generating stress susceptibility.

A number of studies investigating altered gene expression and heritable epigenetic changes have utilised ELMS. The type of maternal care permanently alters gene expression –an example of environmental programming (Meaney and Szyf, 2005). Reduced levels of presynaptic nectin1 cell adhesion molecule in the medial entorhinal cortex lead to an impairment of spatial memory and decreased neuronal plasticity in CA1 (Wu et al., 2022). Decreased expression of the developmental transcription factor *Otx2* in the VTA increases stress susceptibility in adulthood following CSDS (Peña et al., 2017). Site-specific methylation of a glucocorticoid receptor gene promoter in the hippocampus is induced by strong maternal care and this epigenetic change persists into the adulthood, protecting from decreased neurogenesis and structural plasticity that is present in mice that underwent ELMS (Mirescu et al., 2004; Weaver et al., 2004; Fabricius et al., 2008; Kember et al., 2012). Enhanced freezing in adult mice can be prevented by blocking glucocorticoid receptors during adolescence (Arp et al., 2016). Consistent with other models, ELMS rats exhibited a methylation-caused decrease in BDNF levels in the PFC region (Roth et al., 2009). These changes are transmitted to the female offspring which may lead to poor parental care and abusive behaviours that these maltreated females exhibit in toward their offspring (Roth et al., 2009). Interestingly, offspring of the mice that underwent ELMS exhibited depressive-like behaviours even when reared in standard conditions (Franklin et al., 2010). Another group of genes that are consistently epigenetically modulated are serotonin neurotransmission-related genes (Soga et al., 2021). Neonatal stress paradigms cause attenuated efficacy of serotonergic neurotransmission in the hippocampus and raphae nuclei (Lee et al., 2007; Diamantopoulou et al., 2018). However, no changes in either 5-HT_1*A*_ mRNA or serotonin mRNA were found in the hippocampus (Bravo et al., 2014). The DRN displayed downregulated transcription of both serotonin and its receptor 5-HT_1*A*_, whereas the amygdala displayed upregulated transcription of the 5-HT_1*A*_ (Bravo et al., 2014). Dopaminergic neurotransmission is attenuated at the transcriptome level specifically in the VTA of adult male mice that underwent ELMS (Catale et al., 2022). A detailed review of the ELS effect on major neurotransmission systems has been previously published (Marco et al., 2015). Finally, neonatal stress susceptibility was found to be specifically mediated through demethylation of lysine 79 of histone 3 in D2-type medium spiny neurons of NAc, whereas inhibition of this process reversed the behavioural deficits (Kronman et al., 2021).

##### Strengths and weaknesses

These paradigms exhibit relatively high construct and face validity. One criticism of the construct validity certainly lies in the fact that neonatal rodents’ brain development stage is equivalent to that of human foetal brain in the third trimester, despite the major brain development milestones being highly similar (Bayer et al., 1993). The validity parameters will have to be systematically reassessed in the future with respect to high variation in the neonatal protocols which is the major progress-undermining weakness of the paradigm. This is particularly important given that different types of stressors, their predictability, delivery duration, and severity, specifically during early development, can produce opposing results (Cohodes et al., 2021). A meta-analysis of 92 neonatal stress studies found no consistent deleterious effects of these paradigms and noted the methodological differences (Tractenberg et al., 2016). Almost an equal number of studies reported ameliorating and deleterious effects (Tractenberg et al., 2016). Results from different paradigms cannot be compared *prima facie*. Even results obtained by the same paradigm should be examined in the light of the developmental stage at which the stressor is applied as this can produce specific behavioural and neuroanatomical outcomes (Leussis et al., 2012; Freund et al., 2013; van Bodegom et al., 2017). This aligns with human studies which found that different disorders and region-specific changes occur as a function of age and developmental stage (Kaplow and Widom, 2007; Andersen and Teicher, 2008; Schoedl et al., 2010). Particularly important is the establishment of sensitive periods for different paradigms and association of the stressors with experience-dependent and experience-expectant processes (Andersen, 2015).

Importantly, some mild stressors at this developmental stage, such as brief maternal separation that mimics mother leaving to search for food, might induce subsequent stress tolerance and improved coping responses toward future stressors (Papaioannou et al., 2002; Plotsky et al., 2005; Oomen et al., 2010; Russo et al., 2012). Recent findings indicate that predictable neonatal stress induces pro-resilience, whereas unpredictable neonatal stress induces pro-susceptibility (Shi et al., 2021). Consistently human adolescents who experienced mild childhood adversity also exhibit better stress coping (Shapero et al., 2015). Rearing in communal nests with a higher number of pups and mothers can confer resilience to social stress (Branchi et al., 2013). The stress-buffering effects of milder predictable neonatal stress forms remain an important underexplored research venue (Shi et al., 2021). Overall, the usefulness of neonatal paradigms consists of controllable environmental programming which allows for study of (1) interactions between genetic and environmental vulnerability factors, and (2) modulation of adulthood stress coping responses by the ELS (Dich et al., 2015). As seen, neonatal paradigms exhibit excellent construct validity. Face validity is limited by discrepancy in the brain development between humans and rodents. Additional pharmacological studies will be required to confidently assess the predictive validity of this model.

### Chronic restraint stress

Chronic restraint stress (CRS) attempts to model a stressful inescapable situation, without delivering a painful stimulus. The paradigm consists of placing a rodent into a confined tube for 2–6 h over 2–3 weeks (Pollak et al., 2010). CRS lasting less than 2 weeks mostly fails to induce behavioural changes (Wang, 2017). Longer sessions of 24 h induce depressive-like behaviours for more than a month, which are reversible by fluoxetine administration (Chu et al., 2016). Anxiety-like behaviours emerge after 1 week of stress, whereas anhedonic behaviours emerge after 5 weeks of stress (Codeluppi et al., 2021). It has been reported that CRS and CSDS induce similar changes in anhedonic, social-avoidant, and immobile behaviour (Haenisch et al., 2009; Veena et al., 2009; Ulloa et al., 2010; Voorhees et al., 2013; Mao et al., 2022). Furthermore, CRS increases grooming and anxiety-like exploratory deficits, while decreasing social dominance (D’Aquila et al., 2000; Chiba et al., 2012; Park et al., 2018). A recent finding indicates that GABAergic VTA cells are responsible for CRS-induced anhedonic behaviour (Lowes et al., 2021). PFC-dependent working memory and temporal order recognition memory are impaired due to dampened glutamatergic transmission (Mika et al., 2012; Yuen et al., 2012; Park et al., 2018). Like other paradigms, CRS impairs hippocampal neurogenesis, reduces BDNF and glucocorticoid receptor levels, and alters the levels of synaptic plasticity markers (Bravo et al., 2009; Haenisch et al., 2009; Chiba et al., 2012). These changes are reversible by chronic reboxetine and desipramine treatments and preventable by enriched environments (Bravo et al., 2009; Haenisch et al., 2009; Veena et al., 2009). CRS impairs spatial working memory and nesting behaviour through downregulated insulin signalling in the hippocampus (Woo et al., 2018). HPA response is elevated following the first sessions of CRS, but subsides after 3 weeks and does not produce a significant change in CORT levels (Chiba et al., 2012; Voorhees et al., 2013). Basal CORT levels predict the vulnerability to CRS (Kim et al., 2013).

A particular advantage of the CRS is that it induces a depressive-like phenotype in all animals subjected to it (Wang, 2017). Another advantage is that it is a unique model of mild predictable stress. However, predictability and mildness of the paradigm are likely the cause behind reported alleviation of pre-existing depressive- and anxiety-like behaviours (Parihar et al., 2011; Lee et al., 2021). CRS attenuates fear learning and causes locomotor hyperactivity through the facilitation of synaptic plasticity in the anterior cingulate cortex (Ito et al., 2010). Such findings are contrary to the observations in human patients and mice that undergo other stress paradigms (Stetler and Miller, 2011). Recent findings have identified the effects that CRS shares with other paradigms and that mirror those observed in humans (Yuen et al., 2012; Seewoo et al., 2020; Yoo et al., 2022). For example both CRS and CSDS induce elevated firing in the projection from the lateral hypothalamus to the LHb which causes the onset of depressive-like symptoms (Zheng et al., 2022). Additional studies will further confirm the usefulness of this practical and efficient paradigm.

## Concluding remarks

Developing convincing and applicable animal models which faithfully represent specificities of the human condition is a challenging enterprise. Animal models are necessary for understanding neuropathology and the discovery of novel treatments. Despite all the progress made, there is much space for improvement and higher level integration of available findings. Many necessary and sufficient mechanisms driving depressive-like behaviour have been uncovered in rodents. However, serious attempts at integration of the previous findings at the level of associated brain regions, larger circuit formations, and molecular pathways have not yet been made. More than investigating projections driving particular behaviours, complex circuit analysis will be required for further advancement and tools for such endeavour are still emerging. Integration of hundreds of previous studies, alongside necessary close assessment and comparison of behavioural tests and paradigms used, will potentially yield a more coherent narrative about the emergence of depression and mood disorders. Simultaneously, further research in human patients will be needed to validate current behavioural models. By focussing our efforts in both directions, translational convergence between the rodent models and human endophenotypes will improve, accelerating the rate of discovery and understanding of neuropsychiatric disorders.

## Author contributions

AP and DC developed and wrote the manuscript. Both authors contributed to the article and approved the submitted version.

## Abbreviations

BDNF: brain-derived neurotrophic factor
CREB: cAMP response element-binding protein
CRS: chronic restraint stress
CSDS: chronic social defeat stress
CUMS: chronic unpredictable mild stress
CORT: corticotropin
CRH: corticotropin-releasing hormone
CST: Crawley’s sociability test
FST: forced swim test
ELS: early-life stress
ELMS: early-life maternal separation
ICSS: Intracranial self-stimulation
LH: learned helplessness
LHb: lateral habenula
LTP: long-term potentiation
MDD: major depressive disorder
mPFC: medial prefrontal cortex
MSN: medium spiny neurons
NAc: nucleus accumbens
SSRIs: selective serotonin reuptake inhibitors
SI: social interaction test
SDSS: subthreshold CSDS
SPT: sucrose preference test
TST: tail suspension test
TCA: tricyclic antidepressant
VTA: ventral tegmental area
VSDS: vicarious social defeat stress.
